# Building Blocks for Molecular Polygons Based on Platinum Vertices and Polyynediyl Edges

**DOI:** 10.1021/acs.organomet.2c00573

**Published:** 2023-02-13

**Authors:** Brenna K. Collins, Nancy Weisbach, Frank Hampel, Nattamai Bhuvanesh, John A. Gladysz

**Affiliations:** † Department of Chemistry, Texas A&M University, P.O. Box 30012, College Station, Texas 77842-3012, United States; ‡ Institut für Organische Chemie and Interdisciplinary Center for Molecular Materials, Friedrich-Alexander-Universität Erlangen-Nürnberg, Henkestraße 42, Erlangen 91054, Germany

## Abstract

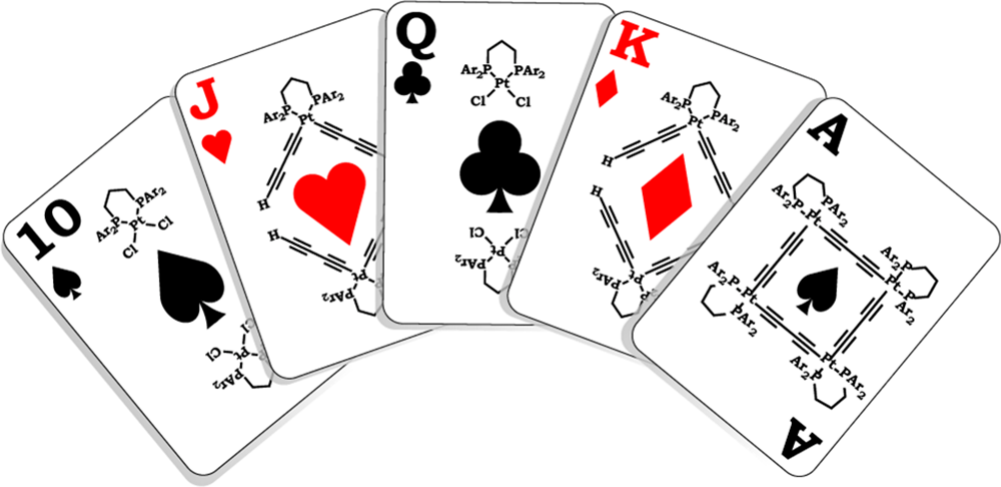

Reactions of Cl_2_P(CH_2_)_3_PCl_2_ and *p*-MgBrC_6_H_4_X (X = **a**/OMe, **b**/O*t*Bu, **c**/*t*Bu, **d**/SiMe_3_) give the diphosphines (*p*-XC_6_H_4_)_2_P(CH_2_)_3_P(*p*-C_6_H_4_X)_2_ (**1a**–**d**; 47–66%). Additions of **1a**,**d** to (COD)PtCl_2_ yield (CH_2_(CH_2_P(*p*-C_6_H_4_X)_2_)_2_)PtCl_2_ (**2a**,**d**; 62–88%), which upon reaction with butadiyne (2 equiv; HNEt_2_/cat. CuI) give (CH_2_(CH_2_P(*p*-C_6_H_4_X)_2_)_2_)Pt((C≡C)_2_H)_2_ (**3a**,**d**; 34–76%). Alternatively, **3a**–**d** can be accessed from *trans*-(*p-*tol_3_P)_2_Pt((C≡C)_2_H)_2_ and **1a**–**d** (30–87%). Reactions of (*p*-tol_3_P)_2_PtCl_2_ and H(C≡C)_2_SiR_3_ (2 equiv, HNEt_2_/cat. CuI; R = Me/Et/*i*Pr) give *trans*-(*p*-tol_3_P)_2_Pt((C≡C)_2_SiR_3_)_2_ (77–95%), and subsequent additions of **1a**,**b**,**d** yield the corresponding adducts (CH_2_(CH_2_P(*p*-C_6_H_4_X)_2_)_2_)Pt((C≡C)_2_SiR_3_)_2_ (R/X = Me/OMe, **5a**; *i*Pr/OMe, **6a**; *i*Pr/O*t*Bu, **6b**; *i*Pr/SiMe_3_, **6d**; 52–95%) and (for **5a**) a luminescent diplatinum byproduct with *trans* Pt((C≡C)_2_SiMe_3_)_2_ units. **5a** and **6b** hydrolyze in the presence of F^–^ to **3a**,**b** (92–93%). Reaction of **2a** and **3a** (HNEt_2_/cat. CuI) affords the Pt_4_C_16_ polygon ([(CH_2_(CH_2_P(*p*-C_6_H_4_OMe)_2_)_2_)Pt(C≡C)_2_]_4_ as an H_2_NEt_2_^+^ Cl^–^ adduct (66%). The ^13^C{^1^H} NMR spectra of **3a**–**d**, **5a**, and **6a**,**b**,**d** feature complex AMXX′ (CPtPP′) spin systems, and simulations allow *J* values to be extracted. The crystal structures of **2a**, **3a**,**b**,**d**, **5a**, and **6a** are determined and analyzed.

## Introduction

Transition metal complexes have been used as building blocks for a number of elegant two- and three-dimensional covalently bound assemblies. Those that can adopt polygonal or polyhedral geometries, with transition metal fragments at the vertices, have received particular attention, and many applications in materials, biological, and catalytic chemistry have been developed.^1−3^ To best access tractable systems, the precursors should (1) be properly encoded geometrically and (2) carry the appropriate solubilizing groups. Species of higher symmetries commonly give more robust crystal lattices, raising melting points per the classical example of toluene versus benzene and lowering solubilities.^4^

In the course of preparing and studying various platinum bis(alkynyl) and diplatinum polyynediyl (Pt(C≡C)_*n*_Pt) complexes,^5−8^ we happened upon reactions that, although not necessarily so designed, appeared to give polygonal species with L_2_Pt corners and polyynediyl edges, as evidenced by mass spectrometry. However, many of these materials, initially generated with 1,2-bis(diphenylphosphino)ethane (dppe) or 1,3-bis(diphenylphosphino)propane (dppp) ligands, were essentially insoluble in all solvents. In particular, those thought to have octatetraynediyl segments (Pt(C≡C)_4_Pt) were the proverbial “brick dust”.

When such problems arise, they can often be solved or ameliorated by incorporating solubilizing substituents such as alkyl groups.^9,10^ For example, we noted earlier that complexes of the formula *trans*,*trans*-(Ar′)(Ar_3_P)_2_Pt(C≡C)_*n*_Pt(PAr_3_)_2_(Ar′) became progressively less soluble as the sp carbon chains were lengthened from *n* = 2 to 4 to 6 for Ar = phenyl. However, analogues with Ar = *p*-tol exhibited much improved solubility levels.^9^

**Scheme 1:**
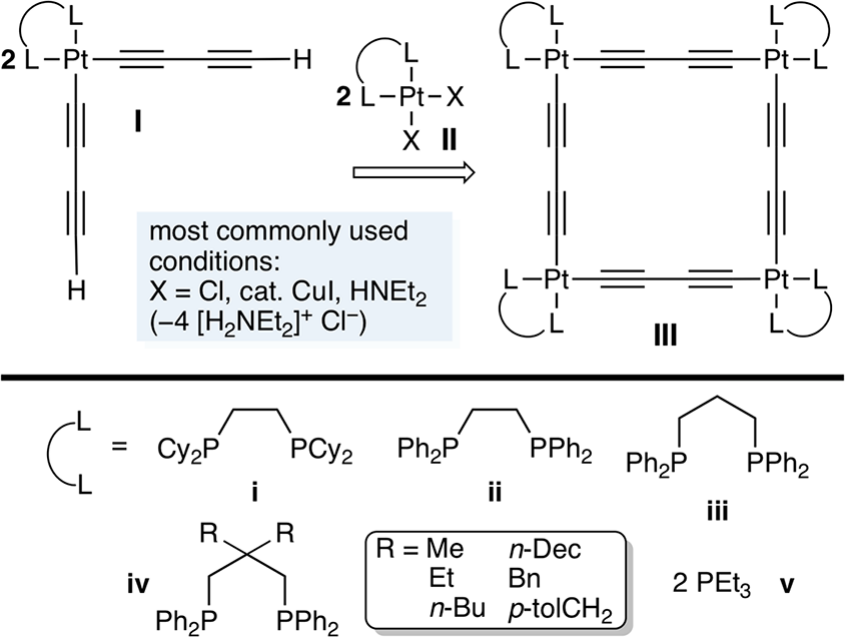
Syntheses of Pt_4_C_16_ Macrocycles (**III**) That Can Adopt Square or Skew Rhombus Geometries

Thus, we set out to prepare building blocks of the formula *cis*-L_2_PtZ_2_ that feature substituted dppp ligands (L_2_). The chelate encodes the *cis* geometry required of corner units. For benchmarking purposes, we sought to first apply such bis(butadiynyl) and dichloride complexes (**I** and **II**, Scheme 1) in the syntheses of the four-sided Pt_4_C_16_ adducts **III**, which have been studied by several research groups.^11−14^ Although no serious solubility issues have arisen in this chemistry, it should be noted that the Pt_4_C_16_ macrocycles have extraordinary affinities for ammonium salts, which are coproduced in most preparative routes. Recently, crystal structures of these supramolecular assemblies have been obtained, together with NMR evidence for their persistence in solution.^11b^

In earlier efforts, we prepared **I**, **II**, and **III** with a variety of backbone-substituted dppp derivatives, Ph_2_PCH_2_CR_2_CH_2_PPh_2_ (**iv**, Scheme 1).^11^ However, additional building blocks with substituted phenyl groups were also sought. Accordingly, in this Article, we report analogous platinum complexes with new ligands of the formula (*p*-XC_6_H_4_)_2_PCH_2_CH_2_CH_2_P(*p*-C_6_H_4_X)_2_ (**1**). The spectroscopic and structural properties of the corresponding dichloride and bis(butadiynyl) complexes are examined in detail, and their viability as precursors to **III** was demonstrated. No portion of this work has been previously communicated.

## Results

### Dichloride and Bis(butadiynyl) Complexes

**Scheme 2:**
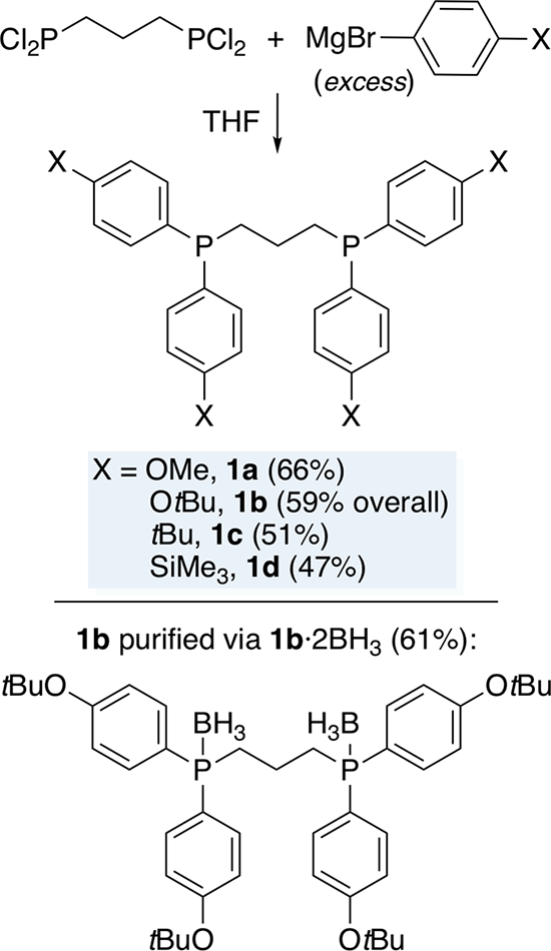
Syntheses of the 1,3-Diphosphines **1**

As shown in Scheme 2, the readily available diphosphine tetrachloride Cl_2_P(CH_2_)_3_PCl_2_^15^ was treated with an excess of the Grignard reagents *p*-MgBrC_6_H_4_X (X = **a**/OMe; **b**/O*t*Bu; **c**/*t*Bu; **d**/SiMe_3_). Workups gave the target ligands **1a**–**d** in 47–66% yields. In the case of **1b**, this involved initial conversion to the bis(borane) adduct **1b**·2BH_3_, which was easier to purify. The borane protecting groups were then removed using excess HNEt_2_.^16^ The phosphines **1a**–**d**, and their complexes below, were characterized by NMR (^1^H, ^13^C{^1^H}, ^31^P{^1^H}) and additional techniques as detailed in the Experimental Section. They could be stored as solids in air at room temperature for weeks, but oxidized much more rapidly in solution. Although **1a** can be found in the literature,^17^ to our knowledge a synthesis has not been described.

**Scheme 3:**
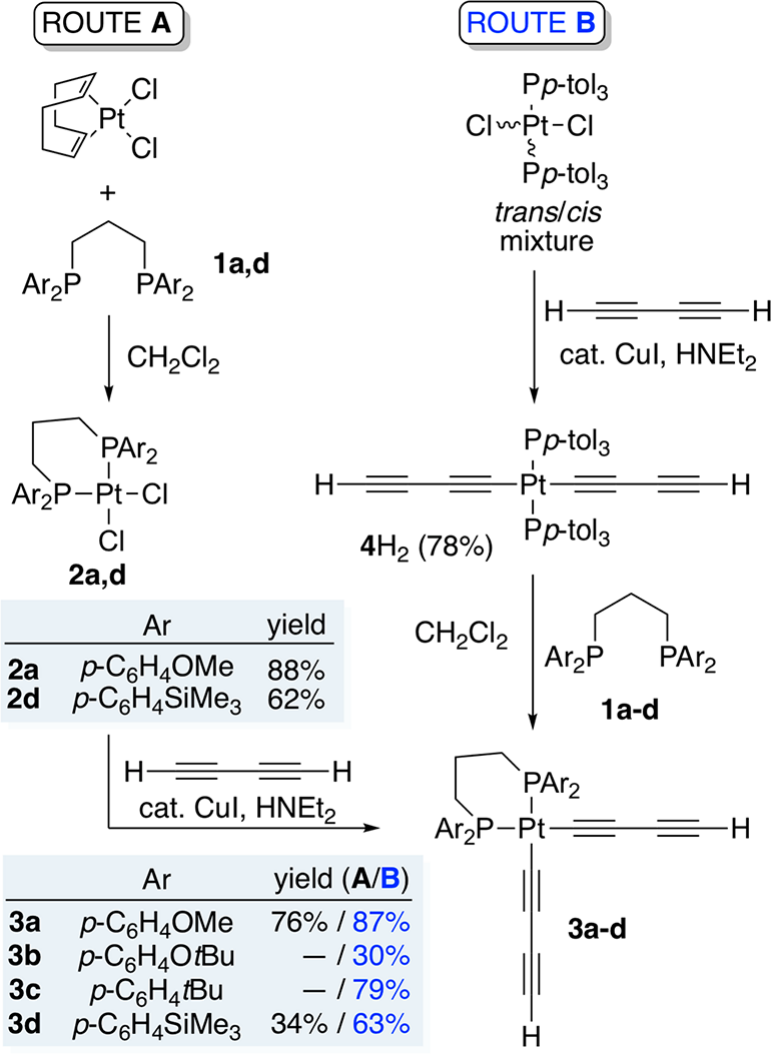
Syntheses of the *cis*-Platinum Complexes **2** and **3**

The 1,3-diphosphines were next elaborated into platinum dichloride and bis(butadiynyl) complexes of the types **I** and **II** (Scheme 1). As shown in Scheme 3, the cyclooctadiene complex (COD)PtCl_2_^18^ was treated with **1a**,**d** (1.01–1.03 equiv). Workups gave the expected dichloride complexes (CH_2_(CH_2_P(*p*-C_6_H_4_X)_2_)_2_)PtCl_2_ (**2a**,**d**) as air- and moisture-stable white solids in 62–88% yields.

Two approaches to **I** were investigated. In line with Scheme 1, platinum chloride complexes commonly react with terminal alkynes and related HC≡C species in the presence of HNEt_2_ (cosolvent) and a catalytic amount of CuI to give adducts with PtC≡C linkages (“Hagihara conditions”).^19^ As depicted in Scheme 3 (“route A”), analogous reactions of **2a**,**d** with excess butadiyne gave the bis(butadiynyl) complexes (CH_2_(CH_2_P(*p*-C_6_H_4_X)_2_)_2_)Pt((C≡C)_2_H)_2_ (**3a**,**d**) in 34–76% yields as white or off-white powders.

In a second approach to **3** (“route B”), the bis(butadiynyl) complex *trans*-(*p*-tol_3_P)_2_Pt((C≡C)_2_H)_2_ (**4**H_2_) was prepared as previously reported^7b^ from (*p*-tol_3_P)_2_PtCl_2_^20^ and butadiyne. As shown in Scheme 3, reactions with the diphosphines **1a**–**d** afforded **3a**–**d** in 30–87% yields. Although **3a**–**d** were quite air- and moisture-stable as solids, they discolored within several hours in solution at room temperature and were more labile than closely related *trans* species such as **4**H_2_. Their NMR spectra are described in detail below, and IR spectra exhibited characteristic ν_C≡C_ (2149–2151 cm^–1^ m) and ν_≡CH_ (3275–3315 cm^–1^ w) bands as summarized in Table S1.

### Trialkylsilylbutadiynyl Complexes

**Scheme 4:**
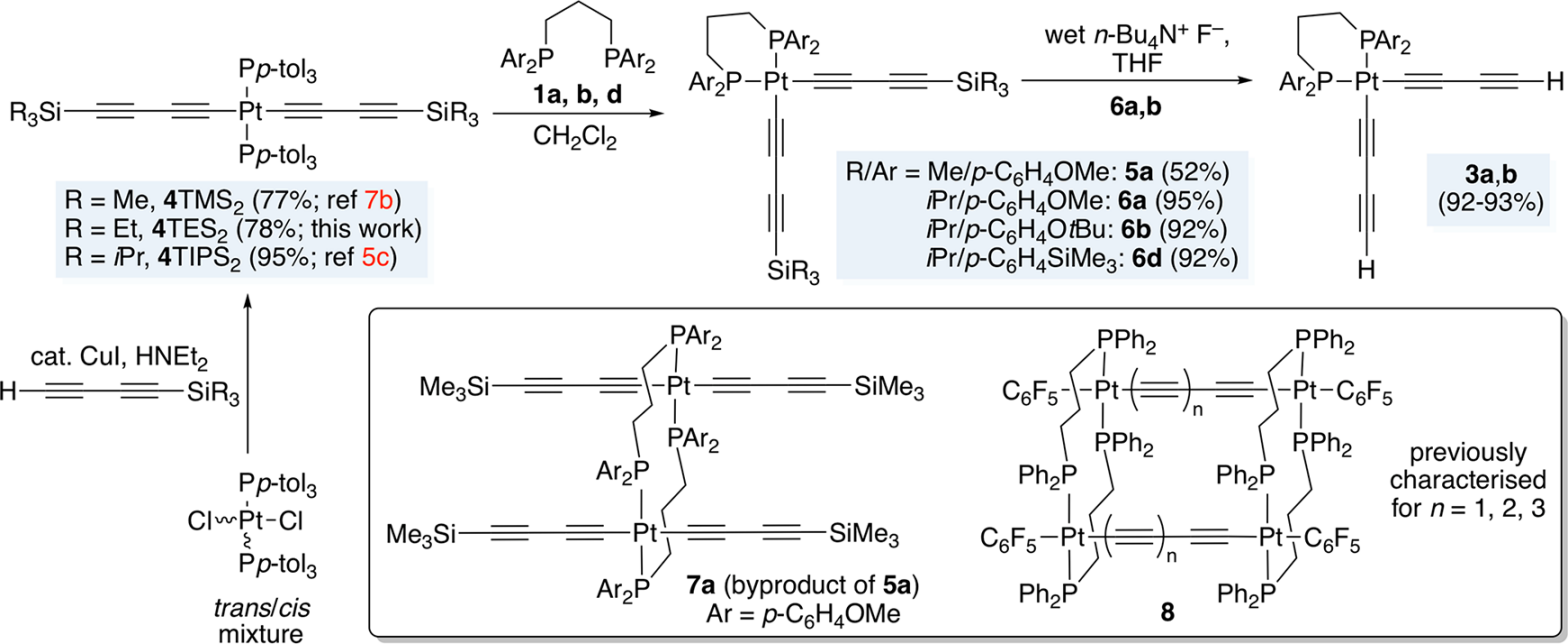
Syntheses of Trialkylsilylbutadiynyl Complexes

Silylated derivatives of **3a**–**d** were also seen as potentially attractive building blocks for **III** or higher homologues. Thus, as shown in Scheme 4, precursors of the formula *trans*-(*p*-tol_3_P)_2_Pt((C≡C)_2_SiR_3_)_2_ (R = Me, **4**TMS_2_; Et, **4**TES_2_; *i*Pr, **4**TIPS_2_) were prepared from (*p*-tol_3_P)_2_PtCl_2_^20^ and the corresponding silylated butadiynes H(C≡C)_2_SiR_3_ under Hagihara conditions. Workups gave **4**TMS_2_, **4**TES_2_, and **4**TIPS_2_ in 77–95% yields. Two of these syntheses have been reported in earlier studies.^5c,7b^

Next, in procedures parallel to those with **4**H_2_ in Scheme 3, various combinations of the bis(trialkylsilylbutadiynyl) complexes and diphosphines **1a**,**b**,**d** were reacted. As shown in Scheme 4, workups gave the desired *cis* monoplatinum adducts (CH_2_(CH_2_P(*p*-C_6_H_4_X)_2_)_2_)Pt((C≡C)_2_SiR_3_)_2_, with the yields higher for the TIPS_2_ systems **6a**,**b**,**d** (92–95%) than the TMS_2_ derivative **5a** (52%). Further reactions of **6a**,**b** and *n*-Bu_4_N^+^F^–^ in wet THF effected desilylation to the bis(butadiynyl) complexes **3a**,**b** in 92–93% yields.

A second product, **7a**, always accompanied **5a**. It was isolated from one run in 10% yield, but NMR yields could be higher. The mass spectrum suggested a formally dimeric diplatinum species. The Pt**C**≡C ^13^C{^1^H} NMR signals were triplets (^2^*J*_CP_), requiring two equivalent phosphorus atoms, as opposed to the complex multiplets found in the *cis* complexes **3a**–**d** and **6a**,**b**,**d** (vide infra). The ^31^P{^1^H} NMR chemical shift and ^1^*J*_PPt_ value (12.0 ppm, 2459 Hz) correlated well with crystallographically characterized PtC≡C species in which two μ-dppe ligands span the same two platinum atoms (i.e., a C≡CPt(PPh_2_CH_2_CH_2_CH_2_PPh_2_)_2_PtC≡C moiety), shown as **8** in Scheme 4.^7a^ The ^13^C{^1^H} NMR chemical shifts of the PCH_2_CH_2_CH_2_P segments in **7a** and **8** were also in excellent agreement (±1.5 ppm).

**Figure 1:**
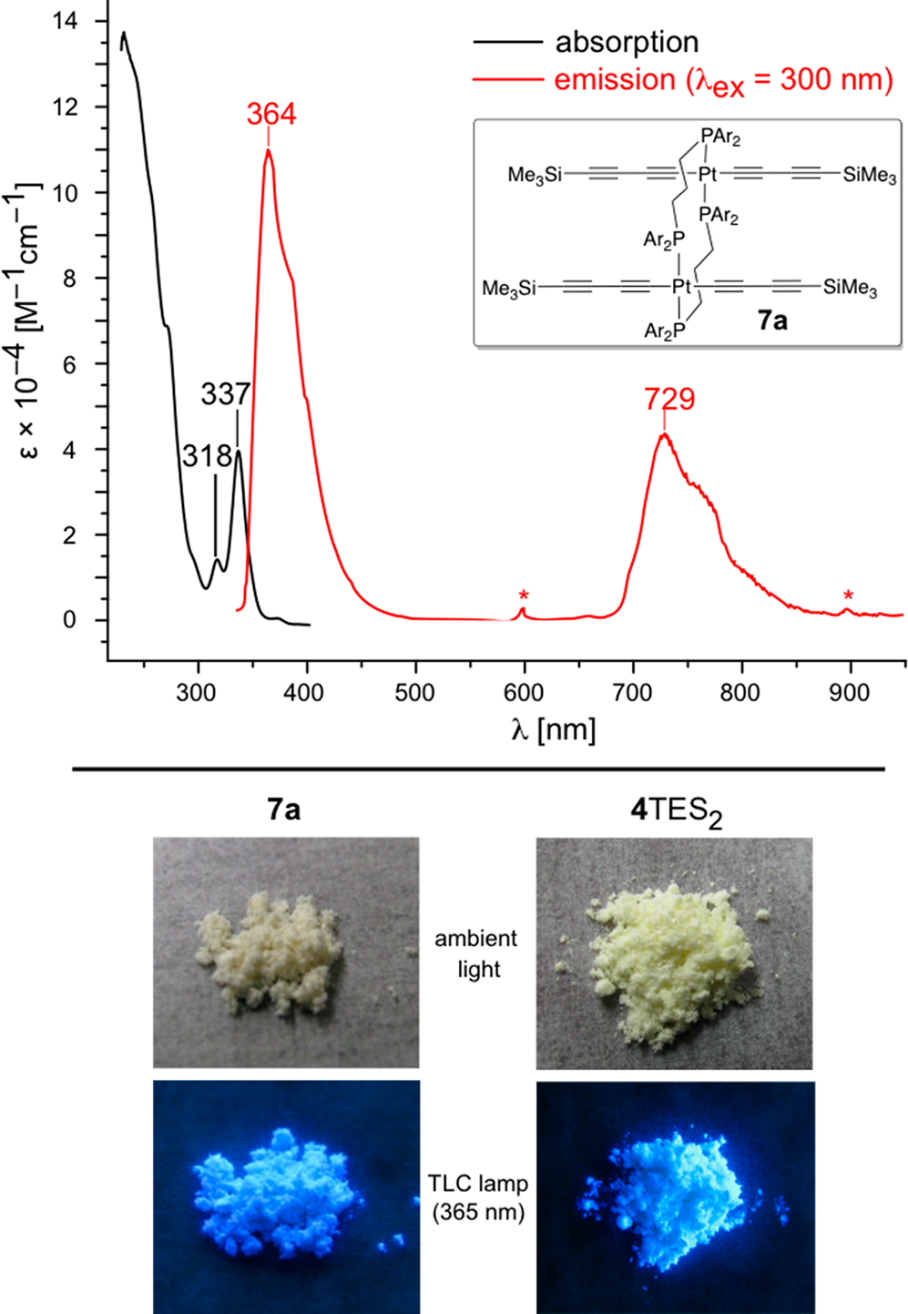
Top: UV–visible (black) and emission (red) spectra of **7a** (CH_2_Cl_2_, ambient temperature; asterisks denote overtones of the excitation wavelength). Bottom: Solid **7a** and **4**TES_2_ under ambient light and under that of a TLC visualization lamp.

Accordingly, **7a** was surmised to be the diplatinum complex *trans*,*trans*-(Me_3_Si(C≡C)_2_)_2_Pt((*p*-MeOC_6_H_4_)_2_P(CH_2_)_3_P(*p*-C_6_H_4_OMe)_2_)_2_Pt((C≡C)_2_SiMe_3_)_2_ (Scheme 4), a structural motif with considerable literature precedent.^21^ As was often seen for complexes with *trans* C≡CPtC≡C linkages,^22^ including **4**TMS_2_, **4**TES_2_, and **4**TIPS_2_ and closely related species,^5c,22a^**7a** luminesced. UV–visible and emission spectra, and related photographic data, are depicted in Figure 1.^22e^ Because no counterparts to **7a** were detected in the TIPS series, further reactions of **4**TMS_2_ and **4**TES_2_, with their less bulky trialkylsilyl groups, were not pursued.

### Application to a Polygonal System

**Scheme 5:**
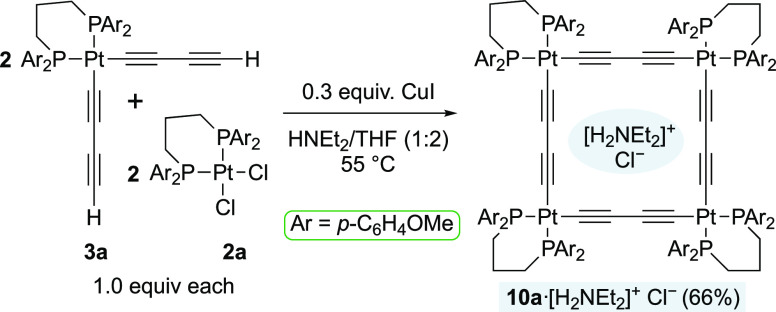
Synthesis of the Polygonal Complex **10a**·[H_2_NEt_2_]^+^Cl^–^

It was sought to establish that the new monoplatinum building blocks were competent partners for the syntheses of Pt_4_C_16_ systems **III**. As illustrated in Scheme 5, **2a** and **3a** were combined in equimolar amounts under Hagihara conditions. After 3 h at 55 °C, a white precipitate was isolated. NMR analyses showed this to be an adduct of the target macrocycle **10a** and the diethylammonium salt coproduct, [(CH_2_(CH_2_P(*p*-C_6_H_4_OMe)_2_)_2_)Pt(C≡C)_2_]_4_·[H_2_NEt_2_^+^ Cl^–^] (**10a**·[H_2_NEt_2_^+^Cl^–^]; 66%). In accord with prior experience with this class of compounds,^11^ the salt could not be removed by washing with a variety of solvents.

The presence of the salt was also evidenced by mass spectrometry and microanalysis (correct N/Cl/H (but not C) values). The nature of the macrocycle/salt interactions has been established in earlier work as briefly summarized below.^11^ Similar reactions were also carried out with **3a**,**c**,**d** and the palladium dppp complex (CH_2_(CH_2_P(C_6_H_5_)_2_)_2_)PdCl_2_.^23^ No well-defined adducts could be isolated under a variety of conditions, although mass spectrometry showed high molecular weight polyplatinum species with mass increments of **3** – H_2_.

### NMR Properties

The ^31^P{^1^H} NMR spectra of the preceding molecules exhibit well-established chemical shift and coupling constant trends, as summarized in Table S1. The ^1^*J*_PPt_ values are characteristic of *cis* complexes,^24^ and notably higher for the dichloride adducts **2a**,**d** (3428–3416 Hz) than for the PtC≡CC≡C species (2191–2213 Hz). As would be intuitively expected, the ^1^H NMR spectra show broad complex multiplets for the PCH_2_CH_2_CH_2_P segments, and no effort has been made to further analyze these. However, ^13^C{^1^H} NMR spectra play key roles in the characterization of carbon-rich metal complexes, so here a deep dive into territory not covered in previous papers was taken.

**Figure 2:**
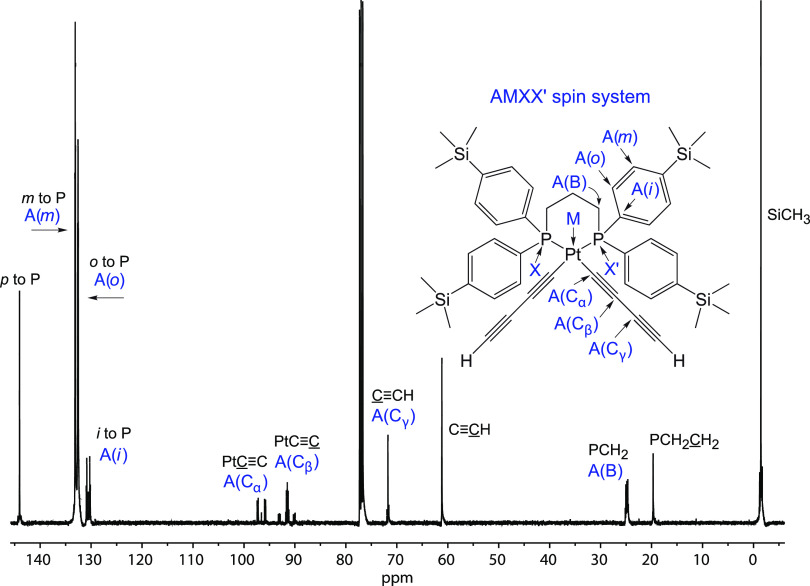
^13^C{^1^H} NMR spectrum of **3d** (CDCl_3_, 125 MHz), which is representative of all newly reported *cis*-bis(butadiynyl) and -bis(trialkylsilylbutadiynyl) complexes.

**Figure 3:**
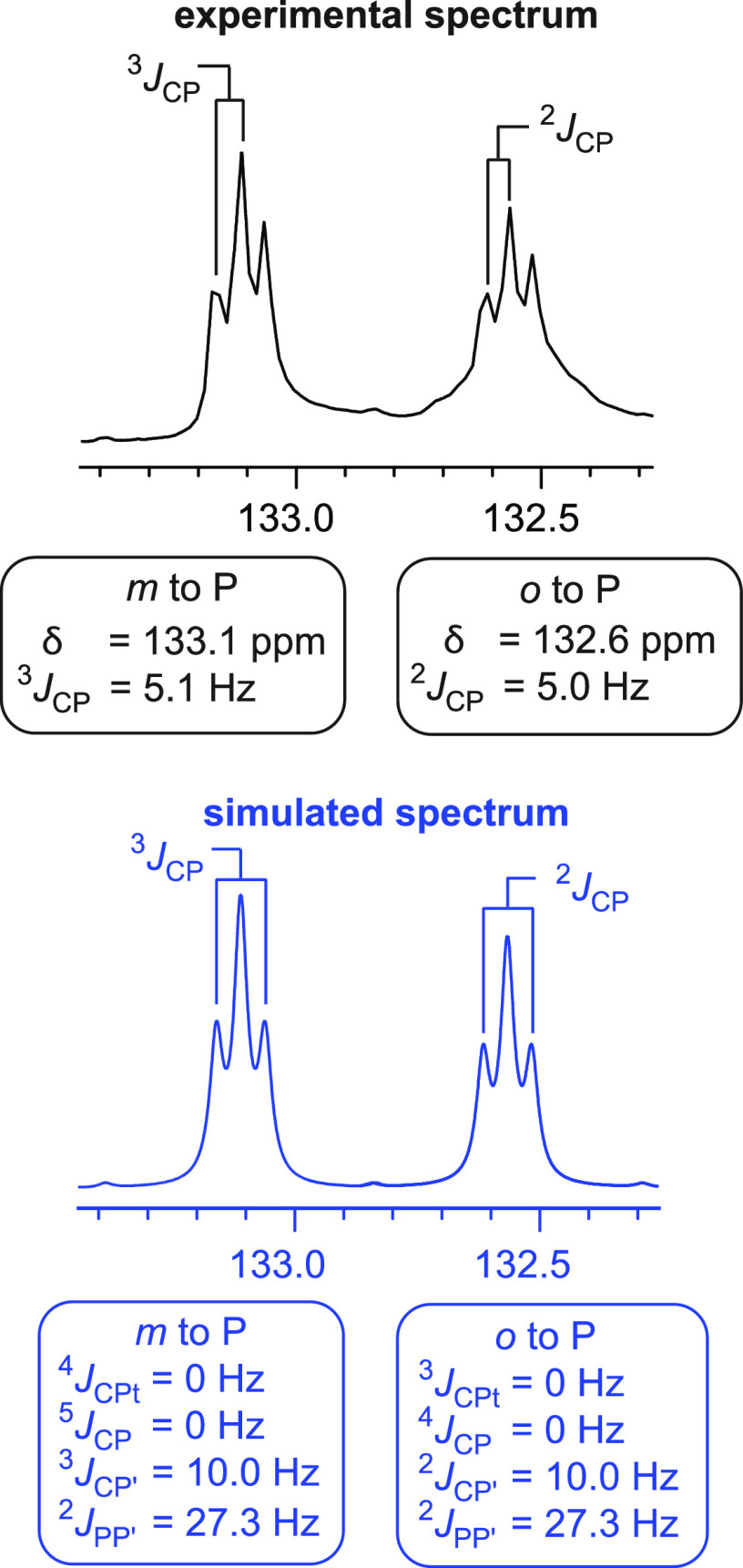
Experimental (black, top) and simulated (blue, bottom) ^13^C{^1^H} NMR spectra (CDCl_3_, 125 MHz) for the carbon atoms *ortho* and *meta* to the phosphorus atoms in **3d**.

**Figure 4:**
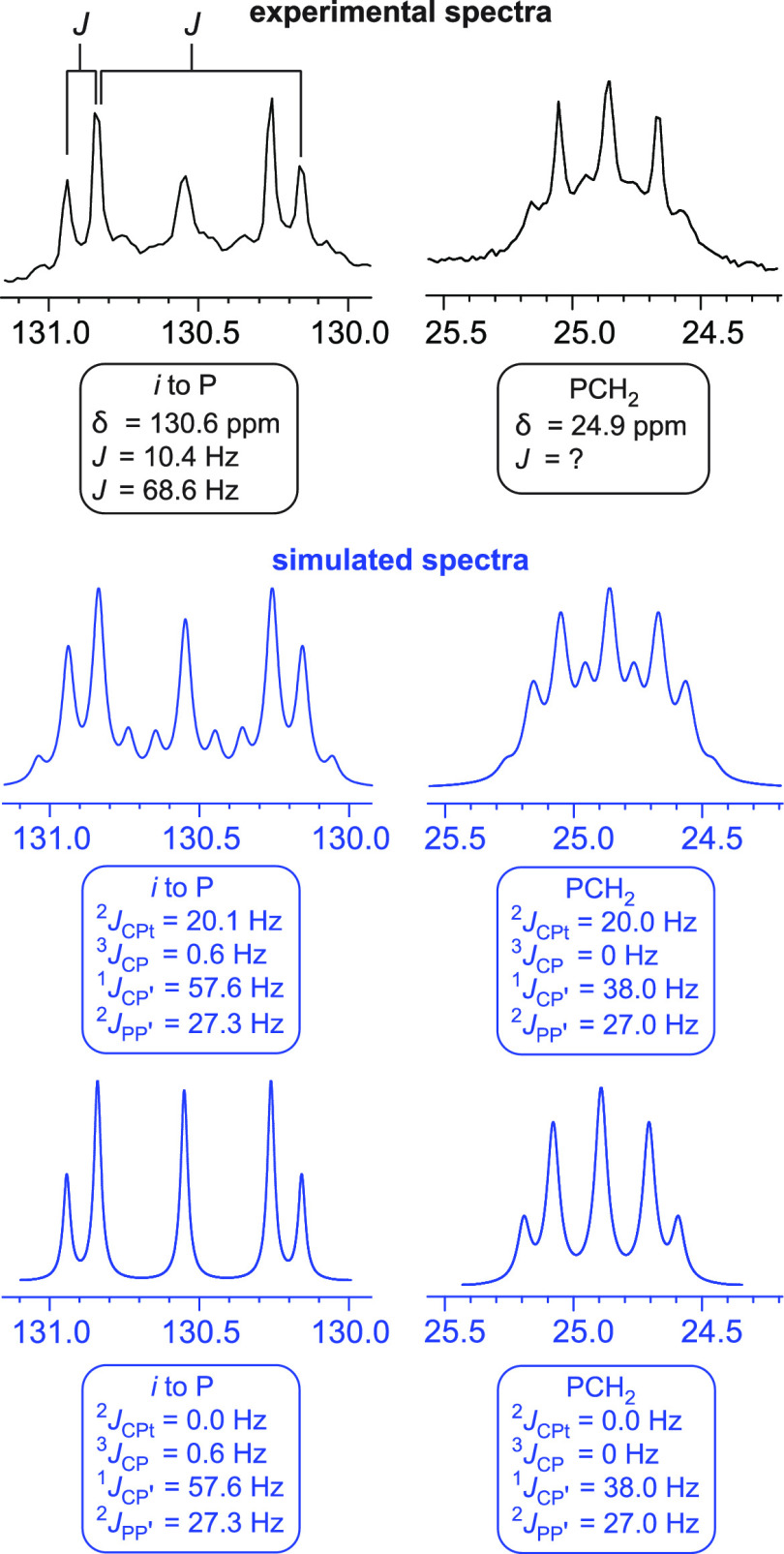
Experimental (black, top) and simulated (blue, middle with ^2^*J*_CPt_ ≠ 0 Hz; blue, bottom with ^2^*J*_CPt_ = 0 Hz) ^13^C{^1^H} NMR spectra (CDCl_3_, 125 MHz) for the *ipso* (left) and PCH_2_ (right) carbon atoms of **3d**.

**Figure 5:**
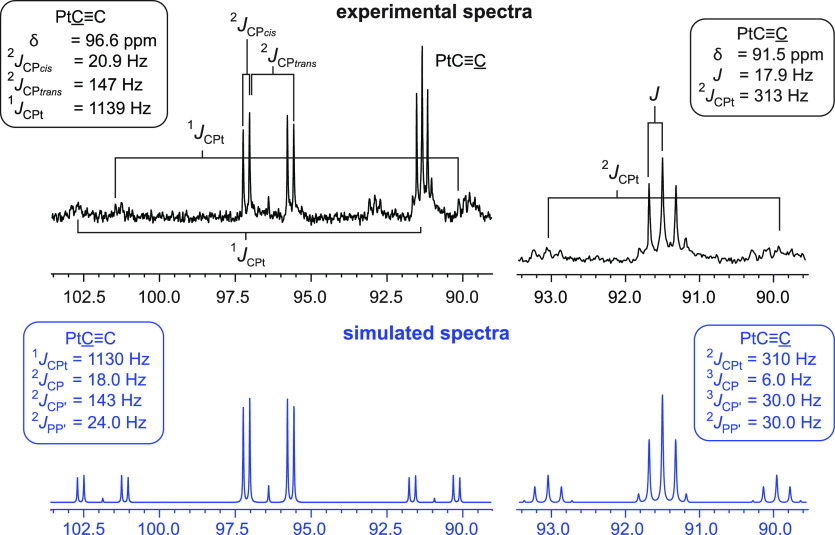
Experimental (black, top) and simulated (blue, bottom) ^13^C{^1^H} NMR spectra (CDCl_3_, 125 MHz) for the PtC≡C carbon atoms of **3d**.

The ^13^C{^1^H} NMR spectrum of a representative *cis*-bis(butadiynyl) complex (**3d**) is depicted in Figure 2. Assignments of the PtC≡CC≡C signals follow from chemical shift and coupling constant relationships established earlier,^5c,25^ and data for all complexes are summarized in Table 1. Data for the *p*-C_6_H_4_X and PCH_2_CH_2_CH_2_P signals are collected in Table S2. For at least one complex in each series, HETCOR, gHMOC, or gHSOC NMR spectra were used to confirm the assignments (e.g., *meta* vs *ortho* carbon signals). Complexes **3a**–**d**, **5a**, and **6a**,**b**,**d** represent AMXX′ (CPtPP′) spin systems, whereby the two chemically equivalent phosphorus atoms couple with each other (^2^*J*_PP′_ ≠ 0). To determine the correct set of coupling constants, the spectrum of **3d** was simulated, as diagrammed in Figures 3–5.^26^

**Table 1:** ^13^C{^1^H} NMR Data (δ/ppm, CDCl_3_; *J* values in Hz) for the C≡CC≡C Segments in the New Platinum Complexes

complex	Pt**C**≡C [^2^*J*_CP*trans*_, ^2^*J*_CP*cis*_, ^1^*J*_CPt_]	PtC≡**C** [*J*,^a^ ^2^*J*_CPt_]	**C**≡CH/Si [^3^*J*_CPt_]	C≡**C**H/Si [^1^*J*_CSi_]
**3a**	96.9 [147, 21.1, −]	91.4 [18.0, −]	71.9 [37.6]	61.2
**3b**	97.3 [147, 21.1, −]	91.3 [18.1, 307]	71.9 [36.1]	61.0
**3c**	97.0 [147, 21.0, −]	91.3 [18.0, 313]	71.9 [39.8]	61.0
**3d**	96.6 [147, 20.9, 1139]	91.5 [17.9, 313]	71.8 [38.8]	61.2
**5a**	100.3 [147, 21.0, 1133]	92.4 [17.8, 309]	92.5 [31.7]	78.2 [87.1]
**6a**	97.9 [147, 21.1, 1133]	93.5 [18.0, 312]	94.2 [34.7]	74.8 [83.2]
**6b**	96.8 [147, 21.2, 1124]	93.3 [17.9, 307]	94.1 [32.8]	74.7 [82.4]
**6d**	96.5 [147, 21.4, 1136]	93.7 [17.8, 313]	94.3 [39.9]	75.0 [82.8]
**7a**	103.6 [15.1, −, −]	92.9 [−, −]^b^	92.5 [−]^b^	76.6 [−]
**10a**	92.5 [164.6, 21.6, −]	97.0 [17.6, −]		

a Multiplet including t with the apparent *J* values indicated; see Figure 5, right.

b This PtC≡**C** versus **C**≡CH assignment is tentative.

As is evident in the expanded Figure 3 (top), the carbon atoms *ortho* and *meta* to phosphorus appear as virtual triplets^27^ similar to those in the spectra of *trans* platinum complexes such as **4**TES_2_. The *J*_CP_ values given in the tables and experimental section represent the difference between two adjacent peaks of the virtual triplet (ca. 5 Hz in Figure 3), that is, “apparent” coupling constants, as always has been carefully footnoted in our papers. However, the simulation (Figure 3, bottom) revealed a true value of 10 Hz, corresponding to the difference between the outer peaks of the virtual triplet.

Figure 4 depicts the observed and simulated spectra for the two types of phosphorus bound carbon atoms, *ipso* aryl (left) and PCH_2_ (right). The latter are reported as multiplets, whereas the former are bookended by a dd pattern, and these apparent *J* values are given in the Experimental Section. However, the simulation confirms that they are not physically meaningful. Importantly, ^195^Pt satellites must be brought into play, as a ^2^*J*_CPt_ value of approximately 20 Hz is required to reproduce the fine structure for each signal (compare the bottom row of spectra in Figure 4 where ^2^*J*_CPt_ is set to 0). The ^2^*J*_CPt_ values for the **C**≡C**Pt**C≡**C** linkages are typically around 260 Hz in *trans* complexes^5a,25^ and around 300 MHz in *cis* complexes (e.g., Figure 5). Such high values distance the ^195^Pt satellites from the “main signal” derived from the magnetically inactive isotopomers. For the sp^3^- and sp^2^-hybridized carbon atoms similarly removed from platinum in **3d**, the ^195^Pt satellites overlap with the main signal, complicating the peak shape.

Figure 5 displays analogous spectra for the Pt**C**≡**C** carbon atoms. Portions of the signals overlap, but all of the Pt**C**≡C signal is represented on the left and the PtC≡**C** signal on the right. At first glance, the former appears to be a doublet of doublets (dd), which would logically be due to *cis* and *trans*^2^*J*_CP_ couplings, for which the *trans*^2^*J*_CP_ value would be expected (from numerous model compounds) to be larger. However, a closer look reveals an additional peak in the center, indicating a higher order multiplet. Nonetheless, the *J*_CP_ values obtained from the simulation (143 Hz, 18.0 Hz) are in good agreement with those from first-order analysis (147 Hz, 20.9 Hz). The experimental spectrum also reveals ^195^Pt satellites with a much poorer signal-to-noise, but the same pattern as the main signal. The ^1^*J*_CPt_ values obtained from the simulation and experiment are similar (1130 Hz vs 1139 Hz).

At first glance, the multiplet for the PtC≡**C** carbon atoms might be taken as a triplet. However, there are clearly outer peaks indicating a higher order multiplet. In the Experimental Section, apparent coupling constants are given (*J* = 17.9 Hz for **3d**) that reflect the difference between adjacent peaks. Importantly, these do not correspond to any coupling constant calculated from the simulation. As with the Pt**C**≡C signals, the measured and simulated ^2^*J*_CPt_ values are almost identical (313 Hz vs 310 Hz).

### Structural Data

**Figure 6:**
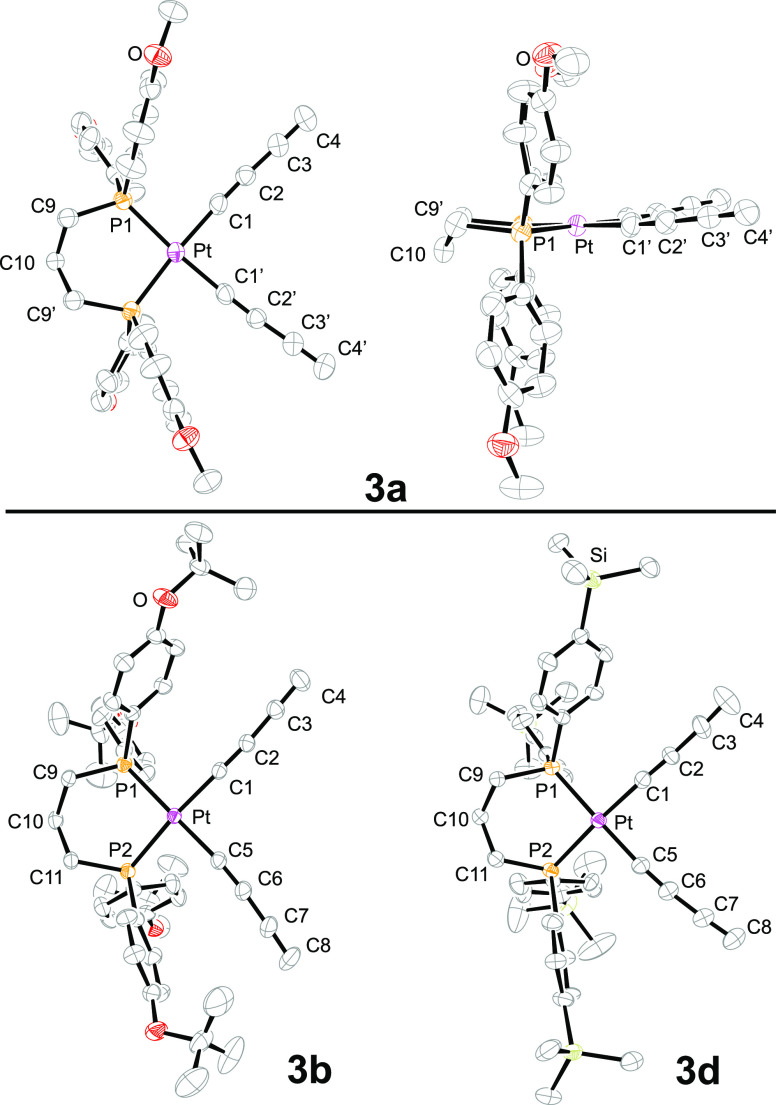
Thermal ellipsoid plots (50% probability level) of the molecular structures of **3a**·(CH_2_Cl_2_), **3b**·(CH_2_Cl_2_)_2_, and **3d**·(CH_2_Cl_2_) with solvent molecules omitted.^28^ For key metrical parameters, see Table 2.

**Figure 7:**
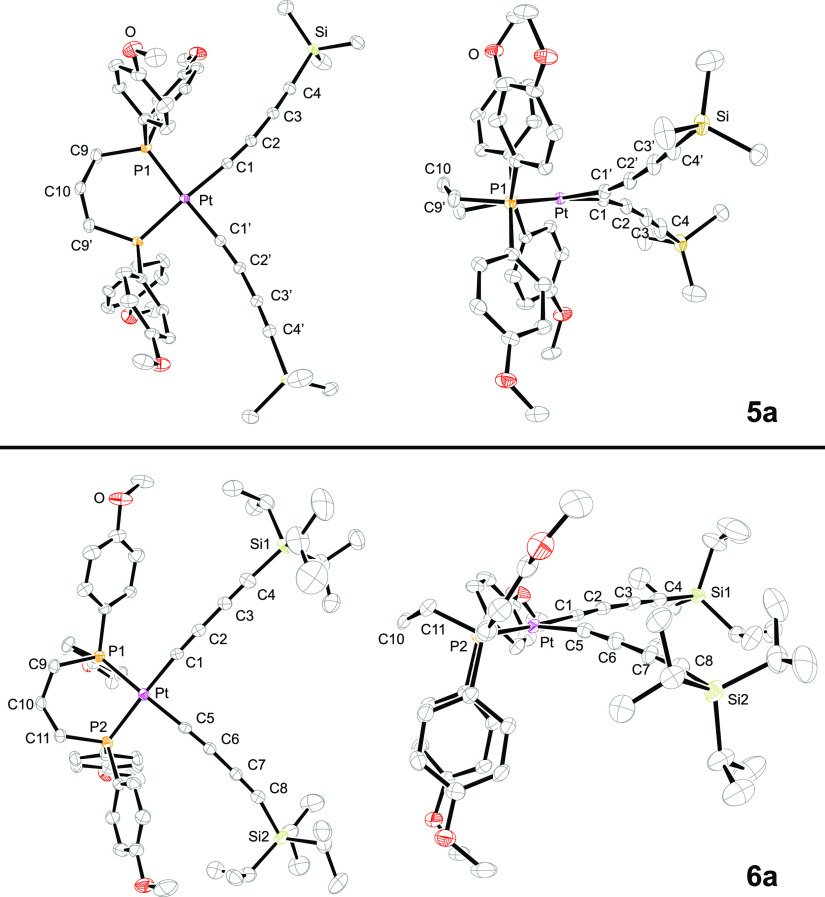
Thermal ellipsoid plots (50% probability level) of the molecular structures of **5a** and **6a**·(Et_2_O) with solvent molecules omitted.^28^ For key metrical parameters, see Table 2.

**Figure 8:**
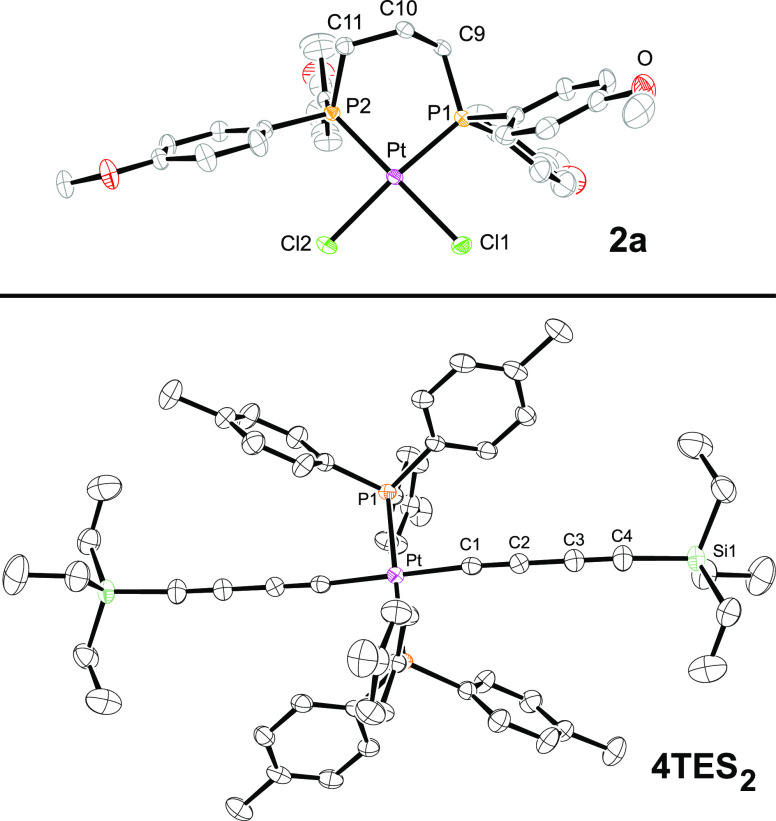
Thermal ellipsoid plots (50% probability level) of the molecular structures of **2a** and **4**TES_2_·(CH_2_Cl_2_) with solvent molecules omitted.^28^ Key bond lengths (Å) and angles (deg): **2a**, Pt–Cl 12.3559(8), Pt–Cl2 2.3567(8), Pt–P1 2.2290(8), Pt–P2 2.2320(8), Cl–Pt–Cl 89.07(3), P1–Pt–P2 92.39(3), Cl1–Pt–P2 179.67(3), Cl2–Pt–P1 172.28(3); **4**TES_2_, Pt–C1 1.986(3), Pt–P 2.3093(6), C1–C2 1.210(4), C2–C3 1.372(4), C3–C4 1.206(4), C4–Si 1.829(3), C1–Pt–C1 180.00, Pt–C1–C2 176.1(2), C1–C2–C3 175.9(3), C2–C3–C4 178.1(3), C3–C4–Si 173.0(3).

In the course of the preceding efforts, crystal structures of seven complexes, or solvates thereof, were determined. These included one *cis* dichloride species (**2a**), five *cis* bis(butadiynyl) or bis(trialkylsilylbutadiynyl) complexes (**3a**,**b**,**d**, **5a**, and **6a**), and the *trans* adduct **4**TES_2_. Data collection and refinements are summarized in the Experimental Section and Tables S3 and S4. The molecular structures are illustrated in Figures 6–8, and additional views of the chelate rings are given in Figure S1.^28^ Key bond lengths and angles for **3a**,**b**,**d**, **5a**, and **6a** are provided in Table 2. The crystal structure of **4**TIPS_2_ has been reported earlier,^5c^ and those of (dppp)Pt((C≡C)_2_H)_2_ and (dppe)Pt((C≡C)_2_H)_2_ have been described by Bruce.^13^ Additional complexes with *cis* Pt(C≡CC≡C)_2_ linkages have also been crystallographically characterized.^29,30^

**Table 2:** Key Crystallographic Distances [Å] and Angles [deg] for **3a**·(CH_2_Cl_2_), **3b**·(CH_2_Cl_2_)_2_, **3d**·(CH_2_Cl_2_), **5a**, and **6a**·(Et_2_O)^28^

	**3a**·(CH_2_Cl_2_)	**3b**·(CH_2_Cl_2_)_2_	**3d**·(CH_2_Cl_2_)	**5a**	**6a**·(Et_2_O)
Pt–C_sp_	1.989(8)	1.996(3)/1.995(3)	1.971(6)/1.988(6)	1.991(2)	1.990(5)/1.991(5)
Pt–P1/Pt–P2	2.2813(19)	2.2956(8)/2.3036(8)	2.3034(15)/2.3096(14)	2.3012(5)	2.2948(13)/2.2942(14)
C1≡C2/C5≡C6	1.221(10)	1.215(4)/1.212(5)	1.215(9)/1.200(9)	1.224(3)	1.207(7)/1.208(7)
C2–C3/C6–C7	1.374(11)	1.377(5)/1.372(5)	1.379(9)/1.408(9)	1.380(3)	1.386(7)/1.386(7)
C3≡C4/C7≡C8	1.185(11)	1.189(5)/1.174(6)	1.193(12)/1.176(10)	1.209(3)	1.191(7)/1.207(7)
avg C≡C	1.203	1.198	1.196	1.216	1.158
Pt···C4/C8	5.763	5.771/5.723	5.751/5.749	5.723	5.767/5.754
C4···C4′/C8	7.675	8.666	7.599	9.175	8.140
C_sp_–Pt–C_sp_	88.6(4)	89.5(12)	88.0(2)	89.9(1)	88.3(2)
Pt–C1–C2/Pt–C5–C6	176.6(7)	177.3(3)/172.7(3)	176.5(6)/175.3(6)	169.6(2)	176.4(4)/168.(5)
C1–C2–C3/C5–C6–C7	176.4(8)	175.3(4)/174.7(4)	177.2(8)/175.0(7)	170.7(2)	175.9(5)/175.9(6)
C2–C3–C4/C6–C7–C8	178.9(9)	178.6(4)/178.3(6)	176.3(10)/176.7(8)	175.0(3)	178.6(6)/177.9(6)
P1–Pt–P2	97.12(9)	94.11(3)	92.08(5)	95.29(3)	90.73(5)
C1–Pt–P2/C5–Pt–P1	175.7(2)	173.2(9)/179.1(1)	176.6(2)/177.4(2)	174.85(6)	175.81(14)
Pt–P1–C9/Pt–P2–C11	116.5	115.9(1)/117.1(1)	115.0(2)/116.1(2)	117.94(8)	115.5(1)/113.9(1)
P1–C9–C10/P2–C11–C10	114.6(6)	113.6(2)/114.8(2)	113.6(4)/115.8(4)	110.9(2)/121.5(2)	113.7(4)/117.9(4)
C9–C10–C11	111.0(9)	113.6(3)	114.4(5)	116.9(3)	115.2(5)
avg C_sp_–C_sp_–C_sp_ and Pt–C_sp_–C_sp_	177.3	176.2	176.2	173.2	174.6

Two of the molecular structures exhibited crystallographic symmetry. With **3a**·(CH_2_Cl_2_) (Figure 6), a mirror plane runs perpendicular to the PPtP plane and includes platinum and the central methylene group of the chelate backbone. In the *trans* complex **4**TES_2_ (Figure 8), there is an inversion center at platinum. Only **5a** exhibited disorder, with the central methylene group (C10) distributed 50:50 over two positions. The limiting forms are related by a *C*_2_ axis, which is coincident with the platinum atom and the midpoint of the P···P vector.

The bond lengths and angles around platinum are very similar to those in related complexes described earlier.^11−14,29,30^ The platinum–phosphorus bonds in the dichloride complex **2a** (2.2290(8) and 2.2320(8) Å, Figure 8) are significantly shorter than those in the *cis* PtC≡C species (2.2923(9) to 2.3096(14) Å, Table 2), consistent with the greater *trans* effect of alkynyl ligands.^31^ As can be seen in Figure S1, some of the chelates exhibit chair-like conformations (e.g., **3b**), others half-chair (e.g., **3a**; see also Figure 6), and others different motifs (**2a**, **5a**) with little analogy in cyclohexane rings (**2a**, **5a**). These are presumably primarily determined by packing forces.

## Discussion

This study has expanded the chessboard of synthetic strategies that can be used to access polygonal Pt_4_C_*x*_ systems such as the Pt_4_C_16_ species **III**. Two series of substituted 1,3-bis(diphenylphosphino)propane (dppp) ligands that enhance the solubilities of platinum adducts are now available, one functionalized on the central carbon atom of the backbone (**iv**, Scheme 1) and the other on the *para* carbon atoms of the aryl rings (**1a**–**d**). The new platinum dichloride and bis(butadiynyl) adducts (Scheme 3) can be applied in existing routes to **III** (Scheme 5) and can potentially be “mixed and matched” with the earlier series to decrease macrocycle symmetry. The bis(trialkylsilylbutadiynyl) complexes (Scheme 4) offer the possibility of additional routes to **III**, as well as enhanced shelf stability and easy conversion to bis(butadiynyl) complexes. Higher homologues of the bis(trialkylsilylbutadiynyl) complexes are readily accessed and are under investigation as precursors to expanded versions of **III**, where exploratory studies have shown that unsubstituted dppp adducts have solubility issues.

Many of the NMR and IR features of the new monoplatinum building blocks have counterparts in the Pt_4_C_16_ complex **10a**·[H_2_NEt_2_^+^Cl^–^] (Tables 1, S1, and S2). Of these, the most important can be seen in the ^13^C{^1^H} NMR spectra in Figures 2–5. All of the complex coupling patterns are manifested in the **10a** core as well as in the similar species **III**-**iv** reported earlier.^11^ Aside from the ^*n*^*J*_CX_ and ^2^*J*_PP′_ values and other insight provided by the simulations, it is comforting to know that the blip often observed within the dd of Pt**C**≡C ^13^C{^1^H} NMR signals is not an impurity but a consequence of the second-order spin system. Also, many structural features of the bis(butadiynyl) and bis(trialkylsilylbutadiynyl) complexes (Figures 6 and 7) are replicated in the nine complexes of the type **III** that have been crystallographically characterized.^11,13,14^ Interestingly, the average C_sp_–Pt–C_sp_ angle calculated from Table 2 (88.9°) is slightly greater than the average for **III** (87.4°, both planar and folded Pt_4_ geometries observed).

**Figure 9:**
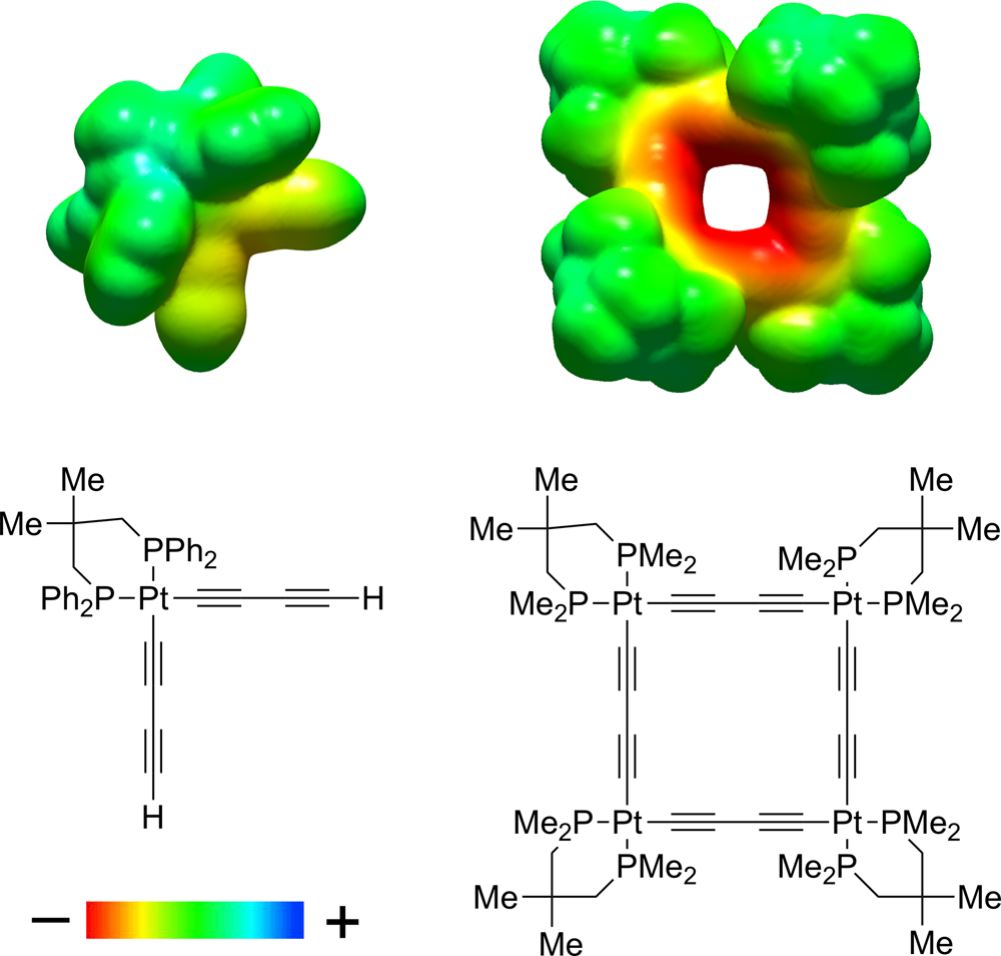
Computed electrostatic surface potential maps for close relatives of **3** and **10a** with modified diphosphine ligands.

Looking ahead, the development of additional routes to **III** and higher homologues is of importance, especially given the difficulties three groups have experienced in removing the H_2_NEt_2_^+^Cl^–^ or other ammonium salt coproducts in Schemes 1 and 5.^11,13,14^ This attraction largely originates from electrostatic surface potentials as mapped in Figure 9.^11a^ The highly negative (red) surface that rings the macrocycle core has no counterpart in the monoplatinum corner units and is furthermore replicated in Pt_3_C_12_ and Pt_5_C_20_ homologues. Crystal structures have established close C≡C/**H**N and NC**H**_2_ contacts,^11b^ and further studies of these phenomena are in progress. To date, only one route appears to sidestep this problem. Bruce reports that treatment of the *cis*-L_2_Pt(C≡CC≡CH)_2_ species **I**-**i**,**ii**,**v** (Scheme 1) with platinum ditriflates *cis*-L_2_Pt(OTf)_2_ at high dilution with NaOAc as the base affords **III**-**i**,**ii**,**v** in 70–73% yields. When amine bases are used, the ammonium triflate coproducts are retained.

The preceding results set the stage for probing access to higher Pt_4_C_24_ and Pt_4_C_32_ homologues of **III**. One impetus among many is the development of corner-extruding reactions and accessing cyclo[*n*]carbon, or cyclic C_*x*_.^32^ Overall, this study highlights how the quest for polygonal and polyhedral systems with metallic vertices often reduces to a “game of corners” with strategic elements not unlike those in similarly named card-deck and playground pastimes.^33^ Progress toward the various targets described above will be detailed in future reports from this laboratory.

## Experimental Section

### General Data

All reactions were conducted under dry inert atmospheres using conventional Schlenk techniques, but workups were carried out in air unless noted. Instrumental methods and other protocols have been detailed in previous papers in this series.^5c,8b,25^ Sources of chemicals are provided in the Supporting Information.

### (*p*-MeOC_6_H_4_)_2_P(CH_2_)_3_P(*p*-C_6_H_4_OMe)_2_ (**1a**)^17^

A Schlenk flask was fitted with an addition funnel, flame-dried under vacuum, and charged with *p*-MgBrC_6_H_4_OMe (1.0 M in THF, 59.3 mL). The funnel was charged with Cl_2_P(CH_2_)_3_PCl_2_ (2.9138 g, 11.853 mmol; see the Supporting Information) dissolved in THF (10 mL), which was added dropwise with stirring. After 17 h, cold saturated aqueous NH_4_Cl (50 mL) was added, and the phases were separated. The aqueous phase was extracted with THF (2 × 50 mL). The combined organic phases were dried (MgSO_4_). The solvents were removed by an oil pump vacuum to give a yellow oil. Crystallization from EtOH/hexanes (−35 °C) gave **1a** as a white powder (4.1449 g, 7.783 mmol, 66%), mp 71–72 °C (open capillary). A sample was further crystallized from methanol (−5 °C) and dried by an oil pump vacuum. Anal. Calcd for C_31_H_34_O_4_P_2_ (532.56): C, 69.92; H, 6.44. Found: C, 70.19; H, 6.36.

NMR (δ/ppm, CDCl_3_): ^1^H (300 MHz) 7.23–7.18 (m, 8H, *o* to P),^34^ 6.76 (d, ^3^*J*_HH_ = 8.0 Hz, 8H, *m* to P),^34^ 3.71 (s, 12H, OC**H**_3_), 2.05–2.00 (m, 4H, PC**H**_2_), 1.55–1.46 (br m, 2H, PCH_2_C**H**_2_); ^13^C{^1^H} (75 MHz) 159.9 (s, *p* to P), 134.0 (d, ^2^*J*_CP_ = 19.8 Hz, *o* to P),^35^ 129.6 (d, ^1^*J*_CP_ = 10.4 Hz, *i* to P), 114.0 (d, ^3^*J*_CP_ = 7.5 Hz, *m* to P),^35^ 55.1 (s, O**C**H_3_), 30.2 (apparent t, *J*_CP_ = 12.2 Hz, P**C**H_2_), 22.3 (t, ^2^*J*_CP_ = 17.2 Hz, PCH_2_**C**H_2_); ^31^P{^1^H} (121 MHz) −19.9 (s).

### (*p*-*t*BuOC_6_H_4_)_2_P(BH_3_)(CH_2_)_3_P(BH_3_)(*p*-C_6_H_4_O*t*Bu)_2_ (**1b**·2BH_3_)

A three-neck flask was fitted with a condenser and an addition funnel, and charged with magnesium (0.437 g, 18.0 mmol) and THF (15 mL). The funnel was charged with *p*-BrC_6_H_4_O*t*Bu (3.456 g, 15.09 mmol) dissolved in THF (10 mL), which was added dropwise over 0.5 h with stirring. The mixture was refluxed (75 °C oil bath). After 6 h, the remaining magnesium was separated, and the solution was transferred to a round-bottom flask fitted with an addition funnel. The funnel was charged with Cl_2_P(CH_2_)_3_PCl_2_ (0.620 g, 2.52 mmol) dissolved in THF (5 mL), which was added dropwise with stirring. After 2 d, cold saturated aqueous NH_4_Cl (20 mL) was added, and the organic phase was separated. The aqueous phase was washed with Et_2_O (3 × 15 mL), and the combined organic phases were dried (Na_2_SO_4_). The solvents were removed by an oil pump vacuum to give crude **1b** (2.501 g) as a highly viscous oil, which was transferred to a Schlenk flask. Et_2_O (40 mL) and BH_3_·SMe_2_ (2.0 M in THF, 6.8 mL, 14 mmol) then were added with stirring. After 50 min, the solvents were removed by rotary evaporation. The residue was chromatographed on a silica gel column (3.5 × 30 cm, packed in hexanes, eluted first with 1:1 v/v CH_2_Cl_2_/hexanes, then with a CH_2_Cl_2_ gradient until pure CH_2_Cl_2_). The solvents were removed from the product containing fractions by rotary evaporation and an oil pump vacuum to give **1b**·2BH_3_ as a white solid (1.115 g, 1.530 mmol, 61% from Cl_2_P(CH_2_)_3_PCl_2_), mp 160–161 °C (open capillary). Anal. Calcd for C_43_H_64_B_2_O_4_P_2_ (728.55): C, 70.89; H, 8.85. Found: C, 71.47; H, 8.73.

NMR (δ/ppm, CDCl_3_): ^1^H (500 MHz) 7.49–7.44 (m, 8H, *o* to P),^34^ 6.99–6.96 (m, 8H, *m* to P),^34^ 2.24–2.19 (m, 4H, PC**H**_2_), 1.75 (br m, 2H, PCH_2_C**H**_2_), 1.35 (s, 36H, C**H**_3_), ∼0.88 (br s, 6H, B**H**_3_); ^13^C{^1^H} (126 MHz) 158.5 (d, ^4^*J*_CP_ = 2.2 Hz, *p* to P), 133.1 (d, ^2^*J*_CP_ = 10.1 Hz, *o* to P),^35^ 123.1 (d, ^3^*J*_CP_ = 10.7 Hz, *m* to P),^35^ 122.1 (d, ^1^*J*_CP_ = 58.8 Hz, *i* to P), 79.3 (s, **C**(CH_3_)), 28.8 (s, **C**H_3_), 27.2 (dd, ^1^*J*_CP_ = 37.1 Hz, ^3^*J*_CP_ = 11.1 Hz, P**C**H_2_), 17.3 (s, PCH_2_**C**H_2_); ^31^P{^1^H} (202 MHz) 13.6 (br s).

### (*p*-(*t*BuOC_6_H_4_)_2_P(CH_2_)_3_P(*p*-C_6_H_4_O*t*Bu))_2_ (**1b**)

A Schlenk flask was charged with **1b**·2BH_3_ (0.953 g, 1.31 mmol), THF (20 mL), and HNEt_2_ (20 mL) and fitted with a condenser. The mixture was refluxed and monitored by TLC and ^31^P{^1^H} NMR. After 20 h (no educt remaining), the solvents were removed by oil pump vacuum. The residue was dissolved in Et_2_O (30 mL) and washed with water (3 × 15 mL). The organic phase was dried (MgSO_4_). The solvents were removed by an oil pump vacuum to give a highly viscous oil. Crystallization from MeOH (−34 °C) gave **1b** as a white solid (0.887 g, 1.27 mmol, 96% or 59% from Cl_2_P(CH_2_)_3_PCl_2_), mp 93–94 °C (open capillary). Anal. Calcd for C_43_H_58_O_4_P_2_ (700.88): C, 73.69; H, 8.34. Found: C, 72.49; H, 8.39.^36^

NMR (δ/ppm, CDCl_3_): ^1^H (500 MHz) 7.24–7.21 (m, 8H, *o* to P),^34^ 6.90 (d, ^3^*J*_HH_ = 8.4 Hz, 8H, *m* to P),^34^ 2.12–2.08 (m, 4H, PC**H**_2_), 1.57 (br m, 2H, PCH_2_C**H**_2_), 1.33 (s, 36H, C**H**_3_); ^13^C{^1^H} (126 MHz) 156.0 (s, *p* to P), 133.4 (d, ^2^*J*_CP_ = 19.6 Hz, *o* to P),^35^ 132.3 (d, ^1^*J*_CP_ = 11.0 Hz, *i* to P), 123.6 (d, ^3^*J*_CP_ = 7.4 Hz, *m* to P),^35^ 78.6 (s, **C**(CH_3_)), 30.3 (apparent t, *J*_CP_ = 12.1 Hz, P**C**H_2_), 28.9 (s, **C**H_3_), 22.5 (t, ^2^*J*_CP_ = 17.3 Hz, PCH_2_**C**H_2_); ^31^P{^1^H} (202 MHz) −19.5 (s).

### (*p*-*t*BuC_6_H_4_)_2_P(CH_2_)_3_P(*p*-C_6_H_4_*t*Bu)_2_ (**1c**)

A Schlenk flask was fitted with an addition funnel and charged with *p*-MgBrC_6_H_4_*t*Bu (2.0 M in Et_2_O; 12 mL, 24 mmol) and THF (20 mL). The funnel was charged with Cl_2_P(CH_2_)_3_PCl_2_ (1.023 g, 4.161 mmol) dissolved in THF (10 mL), which was added dropwise with stirring. After 18 h, during which time a white precipitate formed, cold saturated aqueous NH_4_Cl (20 mL) was added, and the organic phase was separated. The aqueous phase was extracted with THF (3 × 50 mL), and the combined organic phases were dried (Na_2_SO_4_). The solvents were removed by an oil pump vacuum to give a yellowish oil. Crystallization from EtOH/hexanes (−20 °C) gave **1c** as a white solid (1.326 g, 2.082 mmol, 51%), mp 127–128 °C (open capillary). Anal. Calcd for C_43_H_58_P_2_ (636.88): C, 81.09; H, 9.18. Found: C, 80.31; H, 9.30.

NMR (δ/ppm, CDCl_3_): ^1^H (500 MHz) 7.33–7.27 (2 overlapping m, 16H, *o* and *m* to P), 2.16–2.13 (m, 4H, PC**H**_2_), 1.64 (br m, 2H, PCH_2_C**H**_2_), 1.29 (s, 36 H, C**H**_3_); ^13^C{^1^H} (126 MHz) 151.4 (s, *p* to P), 135.1 (d, ^1^*J*_CP_ = 11.1 Hz, *i* to P), 132.5 (d, ^2^*J*_CP_ = 18.6 Hz, *o* to P),^35^ 125.3 (d, ^3^*J*_CP_ = 6.9 Hz, *m* to P),^35^ 34.6 (s, **C**(CH_3_)_3_), 31.3 (s, **C**H_3_), 29.9 (apparent t, *J*_CP_ = 11.9 Hz, P**C**H_2_), 22.8 (t, ^2^*J*_CP_ = 17.5 Hz, PCH_2_**C**H_2_); ^31^P{^1^H} (202 MHz) −19.6 (s).

### (*p*-Me_3_SiC_6_H_4_)_2_P(CH_2_)_3_P(*p*-C_6_H_4_SiMe_3_)_2_ (**1d**)

A three-neck flask was fitted with a condenser and an addition funnel, and charged with magnesium (0.796 g, 32.8 mmol) and THF (5 mL). The funnel was charged with *p*-BrC_6_H_4_SiMe_3_ (5.012 g, 21.87 mmol) dissolved in THF (10 mL), which was added dropwise over 1 h with stirring. The mixture was refluxed (75 °C oil bath). After 3 h, the remaining magnesium was separated, and the solution was transferred to a round-bottom flask fitted with an addition funnel. The funnel was charged with Cl_2_P(CH_2_)_3_PCl_2_ (0.498 g, 2.03 mmol) dissolved in THF (5 mL), which was added dropwise with stirring. After 18 h, during which time a white precipitate formed, cold saturated aqueous NH_4_Cl (15 mL) was added, and the organic phase was separated. The aqueous phase was extracted with THF (2 × 25 mL), and the combined organic phases were dried (Na_2_SO_4_). The solvent was removed by an oil pump vacuum to give a greenish oil. Crystallization from MeOH/hexanes (−20 °C) gave **1d** as a white solid (0.673 g, 0.960 mmol, 47%), mp 120–121 °C (open capillary). Anal. Calcd for C_39_H_58_P_2_Si_4_ (701.18): C, 66.81; H, 8.34. Found: C, 64.53; H, 8.38.^36^

NMR (δ/ppm, CDCl_3_): ^1^H (300 MHz) 7.43–7.33 (2 overlapping m, 16H, *o* and *m* to P), 2.17 (br m, 4H, PC**H**_2_), 1.63 (br m, 2H, PCH_2_C**H**_2_), 0.22 (s, 36 H, Si(C**H**_3_)_3_); ^13^C{^1^H} (75 MHz) 140.8 (s, *p* to P), 139.0 (d, ^1^*J*_CP_ = 13.3 Hz, *i* to P), 133.2 (d, ^3^*J*_CP_ = 6.5 Hz, *m* to P),^35^ 131.9 (d, ^2^*J*_CP_ = 17.9 Hz, *o* to P),^35^ 29.5 (apparent t, *J*_CP_ = 12.0 Hz, P**C**H_2_), 22.7 (s, PCH_2_**C**H_2_), −1.2 (s, ^1^*J*_CSi_ = 52.8 Hz,^37^ Si(**C**H_3_)_3_); ^31^P{^1^H} (121 MHz) −16.6 (s).

### (CH_2_(CH_2_P(*p*-C_6_H_4_OMe)_2_)_2_)PtCl_2_ (**2a**)

A round-bottom flask was charged with (COD)PtCl_2_^18^ (0.211 g, 0.564 mmol), **1a** (0.309 g, 0.580 mmol), and CH_2_Cl_2_ (20 mL) with stirring. After 18 h (a ^31^P{^1^H} NMR spectrum of an aliquot showed no remaining educt), the solvent was removed by rotary evaporation and an oil pump vacuum. The white solid was washed with MeOH and hexanes and dried by an oil pump vacuum to give **2a** as a white powder (0.396 g, 0.496 mmol, 88%) that slightly darkened at 150 °C and became black and liquefied at 304 °C (open capillary). Anal. Calcd for C_31_H_34_Cl_2_O_4_P_2_Pt (798.54): C, 46.63; H, 4.29. Found: C, 47.02; H, 4.52.

NMR (δ/ppm, CDCl_3_): ^1^H (300 MHz) 7.70–7.63 (m, 8H, *o* to P),^34^ 6.89 (d, ^3^*J*_HH_ = 7.7 Hz, 8H, *m* to P),^34^ 3.81 (s, 12H, OC**H**_3_), 2.39 (br m, 4H, PC**H**_2_), 1.98 (br m, 2H, PCH_2_C**H**_2_); ^13^C{^1^H} (126 MHz) 161.7 (s, *p* to P), 135.0 (virtual t, ^2^*J*_CP_ = 5.7 Hz,^38^*o* to P),^35^ 119.7 (m, *J* = 78.4 Hz, *J* = 7.6 Hz, *i* to P),^39^ 114.0 (virtual t, ^3^*J*_CP_ = 6.1 Hz,^38^*m* to P),^35^ 55.3 (s, OC**H**_3_), 25.8–25.3 (m, P**C**H_2_), 18.6 (s, PCH_2_**C**H_2_); ^31^P{^1^H} (121 MHz) −7.0 (s, ^1^*J*_PPt_ = 3428 Hz).^40^

### (CH_2_(CH_2_P(*p*-C_6_H_4_SiMe_3_)_2_)_2_)PtCl_2_ (**2d**)

(COD)PtCl_2_^18^ (0.162 g, 0.432 mmol), **1d** (0.307 g, 0.438 mmol), and CH_2_Cl_2_ (15 mL) were combined in a procedure analogous to that for **2a**. An identical workup gave **2d** as a white powder (0.258 g, 0.267 mmol, 62%) that slightly darkened at 167 °C, became black at 293 °C, but remained solid until 400 °C (open capillary).

NMR (δ/ppm, CDCl_3_): ^1^H (300 MHz) 7.76–7.70 (m, 8H, *o* to P),^34^ 7.52 (d, ^3^*J*_HH_ = 6.6 Hz, 8H, *m* to P),^34^ 2.44 (br s, 4H, PC**H**_2_), 2.00 (br m, 2H, PCH_2_C**H**_2_), 0.26 (s, 36H, Si(C**H**_3_)_3_); ^13^C{^1^H}^41^ 144.7 (s, *p* to P), 133.2 (virtual t, ^2^*J*_CP_ = 5.4 Hz,^38^*m* to P),^35^ 132.7 (virtual t, ^3^*J*_CP_ = 4.8 Hz,^38^*o* to P),^35^ 129.0 (m, *i* to P),^42^ −1.3 (s, Si(**C**H_3_)_3_); ^31^P{^1^H} (121 MHz) −4.1 (s, ^1^*J*_PPt_ = 3416 Hz).^40^

### (CH_2_(CH_2_P(*p*-C_6_H_4_OMe)_2_)_2_)Pt((C≡C)_2_H)_2_ (**3a**)

(A) A round-bottom flask was charged with *trans*-(*p*-tol_3_P)_2_Pt((C≡C)_2_H)_2_ (**4**H_2_;^7b^ 0.207 g, 0.230 mmol), **1a** (0.130 g, 0.244 mmol), and CH_2_Cl_2_ (20 mL) with stirring. After 1.5 h (a ^31^P{^1^H} NMR spectrum of an aliquot showed no remaining educt), the solvent was removed by rotary evaporation, and the brown solid was dried by an oil pump vacuum. The residue was chromatographed on an alumina column (2.5 × 30 cm, eluted first with 1:1 v/v CH_2_Cl_2_/hexanes, then with CH_2_Cl_2_). The solvents were removed from the product containing fractions by rotary evaporation and an oil pump vacuum to give **3a** as a white powder (0.165 g, 0.200 mmol, 87%), which slightly darkened at 115 °C, turned black at 138 °C, and remained solid until 400 °C (open capillary). (B) A round-bottom flask was charged with **2a** (0.369 g, 0.462 mmol), CuI (0.012 g, 0.063 mmol), HNEt_2_ (10 mL), and DMF (30 mL). The mixture was cooled to −45 °C (acetonitrile/CO_2_), and butadiyne^43^ (0.46 g, 9.3 mmol in 2.3 mL THF) was added with stirring. After 0.5 h, the cooling bath was removed. After 0.5 h, the solvents were removed by oil pump vacuum. The residue was suspended in water (30 mL) and stirred for 1 h. The solid was isolated by filtration and washed with EtOH. The remaining solid was extracted with CH_2_Cl_2_, and the black extract was filtered through a pad of Celite. The brown filtrate was concentrated to 10 mL, and hexanes (30 mL) were added. The precipitate was isolated by filtration and dried by an oil pump vacuum to give **3a** as an off-white powder (0.289 g, 0.350 mmol, 76%). A sample was precipitated from DMF/water and similarly dried. Anal. Calcd for C_39_H_36_O_4_P_2_Pt (825.74): C, 56.73; H, 4.39. Found: C, 56.98; H, 4.11. (C) A round-bottom flask was charged with **6a** (see below; 0.080 g, 0.070 mmol) and THF (40 mL). Wet *n*-Bu_4_N^+^F^–^ (1.0 M in THF, 5 wt % water; 0.10 mL, 0.10 mmol) then was added with stirring. After 45 min (TLC showed no remaining educt), the solvent was removed by rotary evaporation and oil pump vacuum. The residue was dissolved in 2:1 v/v CH_2_Cl_2_/hexanes. The sample was filtered through a pad of silica gel (5 × 4 cm), which was sequentially rinsed with 2:1 v/v CH_2_Cl_2_/hexanes, 1:2 v/v ethyl acetate/hexanes, and 2:1 v/v ethyl acetate/hexanes. The solvents were removed from the product containing fractions by rotary evaporation and an oil pump vacuum to give **3a** as an off-white powder (0.054 g, 0.065 mmol, 93%).

NMR (δ/ppm, CDCl_3_): ^1^H (300 MHz) 7.56–7.52 (m, 8H, *o* to P),^34^ 6.85 (d, ^3^*J*_HH_ = 7.9 Hz, 8H, *m* to P),^34^ 3.79 (s, 12H, OC**H**_3_), 2.39–2.35 (m, 4H, PC**H**_2_), 1.93 (br m, 2H, PCH_2_C**H**_2_), 1.63 (s, 2H, C≡C**H**); ^13^C{^1^H} (126 MHz) 161.5 (s, *p* to P), 135.0 (virtual t, ^3^*J*_CP_ = 6.1 Hz,^38^*o* to P),^35^ 121.5 (m, *J* = 73.2 Hz, *J* = 9.8 Hz, *i* to P),^39^ 114.0 (virtual t, ^3^*J*_CP_ = 5.9 Hz,^38^*m* to P),^35^ 96.9 (dd, ^2^*J*_CP*trans*_ = 147 Hz, ^2^*J*_CP*cis*_ = 21.1 Hz, Pt**C**≡C), 91.4 (m, *J* = 18.0 Hz, PtC≡**C**),^44^ 71.9 (s, ^3^*J*_CPt_ = 37.6 Hz,^40^**C**≡CH), 61.2 (s, C≡**C**H), 55.3 (s, O**C**H_3_), 26.1–25.6 (m, P**C**H_2_), 19.6 (s, PCH_2_**C**H_2_); ^31^P{^1^H} (202 MHz) −9.7 (s, ^1^*J*_PPt_ = 2211 Hz).^40^ IR (powder film, cm^–1^) 3275 (w, ν_≡CH_), 2149 (m, ν_C≡C_).

### (CH_2_(CH_2_P(*p*-C_6_H_4_O*t*Bu)_2_)_2_)Pt((C≡C)_2_H)_2_ (**3b**)

(A) A round-bottom flask was charged with **4**H_2_^7b^**(**0.406 g, 0.450 mmol) and CH_2_Cl_2_ (20 mL). A solution of **1b** (0.4493 g, 0.641 mmol)^45^ in CH_2_Cl_2_ (10 mL) was added with stirring. After 2 h (TLC showed no remaining educt), the solvent was removed by an oil pump vacuum. The residue was chromatographed on a silica gel column (3 × 30 cm, packed in hexanes, sequentially eluted with 1:1 v/v CH_2_Cl_2_/hexanes, 1:3 v/v acetone/hexanes, and 2:5 v/v acetone/hexanes). The solvents were removed from the product containing fractions by rotary evaporation and an oil pump vacuum to give **3b** as a tan powder (0.132 g, 0.133 mmol, 30%).^45^ (B) A round-bottom flask was charged with **6b** (see below; 0.098 g, 0.075 mmol) and THF (40 mL). Wet *n*-Bu_4_N^+^F^–^ (1.0 M in THF, 5 wt % water, 0.10 mL, 0.10 mmol) then was added with stirring. After 55 min (TLC showed no remaining educt), the solvent was removed by rotary evaporation and an oil pump vacuum. The residue was chromatographed on a silica gel column (2.5 cm × 25 cm, packed in hexanes, eluted with 1:5 v/v ethyl acetate/hexanes, then with an ethyl acetate gradient until 17:20 v/v ethyl acetate/hexanes). The solvents were removed from the product containing fractions by rotary evaporation and an oil pump vacuum to give **3b** as an off-white powder (0.069 g, 0.069 mmol, 92%).

NMR (δ/ppm, CDCl_3_): ^1^H (500 MHz) 7.49–7.45 (m, 8H, *o* to P),^34^ 6.91 (d, ^3^*J*_HH_ = 7.8 Hz, 8H, *m* to P),^34^ 2.39 (br m, 4H, PC**H**_2_), 1.92 (br m, 2H, PCH_2_C**H**_2_), 1.53 (s, 2H, ≡C**H**), 1.33 (s, 36H, C**H**_3_); ^13^C{^1^H} (126 MHz) 158.0 (s, *p* to P), 134.1 (virtual t, ^3^*J*_CP_ = 5.7 Hz,^38^*o* to P),^35^ 123.5 (m, *J* = 72.2 Hz, *J* = 10.1 Hz, *i* to P),^39^ 122.9 (virtual t, ^3^*J*_CP_ = 5.8 Hz,^38^*m* to P),^35^ 97.3 (dd, ^2^*J*_CP*trans*_ = 147 Hz, ^2^*J*_CP*cis*_ = 21.1 Hz, Pt**C**≡C), 91.3 (m, *J* = 18.1 Hz,^44^^2^*J*_CPt_ = 307 Hz,^40^ PtC≡**C**), 79.4 (s, **C**(CH_3_)_3_), 71.9 (s, ^3^*J*_CPt_ = 36.1 Hz,^40^**C**≡CH), 61.0 (s, C≡**C**H), 28.9 (s, CH_3_), 25.8–25.5 (m, P**C**H_2_), 19.4 (s, PCH_2_**C**H_2_); ^31^P{^1^H} (202 MHz) −9.6 (s, ^1^*J*_PPt_ = 2202 Hz).^40^ IR (powder film, cm^–1^) 3291 (w, ν_≡CH_), 2151 (m, ν_C≡C_).

### (CH_2_(CH_2_P(*p*-C_6_H_4_*t*Bu)_2_)_2_)Pt((C≡C)_2_H)_2_ (**3c**)

A round-bottom flask was charged with **4**H_2_^7b^ (0.850 g, 0.942 mmol), **1c** (0.607 g, 0.953 mmol), and CH_2_Cl_2_ (90 mL) with stirring. After 4 h (a ^31^P{^1^H} NMR spectrum showed no remaining educt), the solvent was removed by rotary evaporation. The solid was dried by an oil pump vacuum and chromatographed on an alumina column (2.5 × 30 cm, first eluted with 1:1 v/v CH_2_Cl_2_/hexanes, then 5:3 v/v with CH_2_Cl_2_/hexanes). The solvent was removed from the product containing fractions by rotary evaporation and an oil pump vacuum to give **3c** as a white powder (0.694 g, 0.746 mmol, 79%) that slightly darkened at 119 °C, became black at 234 °C, and remained solid at 400 °C (open capillary). Anal. Calcd for C_51_H_60_P_2_Pt (730.07): C, 65.86; H, 6.50. Found: C, 65.56; H, 6.61.

NMR (δ/ppm, CDCl_3_): ^1^H (300 MHz) 7.56–7.50 (m, 8H, *o* to P),^34^ 7.34 (d, ^3^*J*_HH_ = 7.6 Hz, 8H, *m* to P),^34^ 2.44 (br s, 4H, PC**H**_2_), 1.97 (br m, 2H, PCH_2_C**H**_2_), 1.55 (s, 2H, C≡C**H**), 1.28 (s, 36H, C**H**_3_); ^13^C{^1^H} (101 MHz) 154.0 (s, *p* to P), 133.3 (virtual t, ^2^*J*_CP_ = 5.5 Hz,^38^*o* to P),^35^ 126.9 (m, *J* = 70.7 Hz, *J* = 10.3 Hz, *i* to P),^39^ 125.3 (virtual t, ^3^*J*_CP_ = 5.5 Hz,^38^*m* to P),^35^ 97.0 (dd, ^2^*J*_CP*trans*_ = 147 Hz, ^2^*J*_CP*cis*_ = 21.0 Hz, Pt**C**≡C), 91.3 (m, *J* = 18.0 Hz,^44^^2^*J*_CPt_ = 313 Hz,^40^ PtC≡**C**), 71.9 (s, ^3^*J*_CPt_ = 39.8 Hz,^40^**C**≡CH), 61.0 (s, C≡**C**H), 34.7 (s, **C**(CH_3_)_3_), 31.0 (s, **C**H_3_), 25.5–24.8 (m, P**C**H_2_), 19.6 (s, PCH_2_**C**H_2_); ^31^P{^1^H} (121 MHz) −9.0 (s, ^1^*J*_PPt_ = 2196 Hz).^40^ IR (powder film, cm^–1^) 3312 (m, ν_≡CH_), 2151 (s, ν_C≡C_).

### (CH_2_(CH_2_P(*p*-C_6_H_4_SiMe_3_)_2_)_2_)Pt((C≡C)_2_H)_2_ (**3d**)

(A) **4**H_2_^7b^ (0.316 g, 0.350 mmol), **1d** (0.271 g, 0.386 mmol), and CH_2_Cl_2_ (20 mL) were combined in a procedure analogous to that for **3c**. An identical workup gave **3d** as a white powder (0.221 g, 0.222 mmol, 63%) that darkened at 139 °C, became black at 194 °C, and remained solid at 400 °C (open capillary). Anal. Calcd for C_47_H_60_P_2_PtSi_4_ (994.37): C, 56.77; H, 6.08. Found: C, 56.57; H, 6.25. (B) A round-bottom flask was charged with **2d** (0.213 g, 0.220 mmol), CuI (0.011 g, 0.058 mmol), and HNEt_2_ (40 mL). The mixture was cooled to −45 °C (acetonitrile/CO_2_), and butadiyne^43^ (0.22 g, 4.4 mmol in 1.1 mL of THF) was added with stirring. After 1 h, the cooling bath was removed. After 1 h, the solvents were removed by an oil pump vacuum. The residue was suspended in water (15 mL) and stirred for 30 min. The solid was isolated by filtration and washed with water and EtOH. The remaining solid was extracted with CH_2_Cl_2_, and the black extract was filtered through a pad of Celite. The brown filtrate was concentrated to 10 mL, and hexanes (30 mL) were added. The precipitate was isolated by filtration and dried by an oil pump vacuum to give **3d** as an off-white powder (0.075 g, 0.075 mmol, 34%).

NMR (δ/ppm, CDCl_3_): ^1^H (300 MHz) 7.61–7.55 (m, 8H, *o* to P),^34^ 7.48 (d, ^3^*J*_HH_ = 6.4 Hz, 8H, *m* to P),^34^ 2.48 (br m, 4H, PC**H**_2_), 2.01 (br m, 2H, PCH_2_C**H**_2_), 1.55 (s, 2H, ≡C**H**), 0.25 (s, 36 H, C**H**_3_); ^13^C{^1^H} (100 MHz) 144.1 (s, *p* to P), 133.1 (virtual t, ^3^*J*_CP_ = 5.1 Hz,^38^*m* to P),^35^ 132.6 (virtual t, ^2^*J*_CP_ = 5.0 Hz,^38^*o* to P),^35^ 130.6 (m, *J* = 68.6 Hz, *J* = 10.4 Hz, *i* to P),^39^ 96.6 (dd, ^2^*J*_CP*trans*_ = 147 Hz, ^2^*J*_CPcis_ = 20.9 Hz, ^1^*J*_CPt_ = 1139 Hz,^40^ Pt**C**≡C), 91.5 (m, *J* = 17.9 Hz,^44^^2^*J*_CPt_ = 313 Hz,^40^ PtC≡**C**), 71.8 (s, ^3^*J*_CPt_ = 38.8 Hz,^40^**C**≡CH), 61.2 (s, C≡**C**H), 25.2–24.6 (m, P**C**H_2_), 19.7 (s, PCH_2_**C**H_2_), −1.5 (s, ^1^*J*_CSi_ = 52.5 Hz,^37^ Si(**C**H_3_)_3_); ^31^P{^1^H} (121 MHz) −6.6 (s, ^1^*J*_PPt_ = 2191 Hz).^40^ IR (powder film, cm^–1^) 3315/3289 (w/w, ν_≡CH_), 2151 (m, ν_C≡C_).

### (CH_2_(CH_2_P(*p*-C_6_H_4_OMe)_2_)_2_)Pt((C≡C)_2_SiMe_3_)_2_ (**5a**) and *trans*,*trans*-(Me_3_Si(C≡C)_2_)_2_Pt((*p*-MeOC_6_H_4_)_2_P(CH_2_)_3_P(*p*-C_6_H_4_OMe)_2_)_2_Pt((C≡C)_2_SiMe_3_)_2_ (**7a**)

A round-bottom flask was charged with **4**TMS_2_^7b^ (0.300 g, 0.286 mmol), **1a** (0.178 g, 0.334 mmol), and CH_2_Cl_2_ (30 mL) with stirring. After 2 h, the solvent was removed by rotary evaporation. The brown solid was dried by an oil pump vacuum and chromatographed on a silica gel column (3.5 × 30 cm, packed in hexanes eluted first with hexanes, then with a CH_2_Cl_2_ gradient until pure CH_2_Cl_2_). The solvents were removed from the product containing fractions by rotary evaporation and an oil pump vacuum to give **5a** as a white powder (0.144 g, 0.148 mmol, 52%) and **7a** as an off-white powder (0.027 g, 0.014, 10%).

For **5a**, the powder slightly darkened at 142 °C and became black and liquefied at 231 °C (open capillary). Anal. Calcd for C_45_H_52_O_4_P_2_PtSi_2_ (970.11): C, 55.71; H, 5.40. Found: C, 55.99; H, 5.53. NMR (δ/ppm, CDCl_3_): ^1^H (500 MHz) 7.51–7.47 (m, 8H, *o* to P),^34^ 6.82 (d, ^3^*J*_HH_ = 7.7 Hz, 8H, *m* to P),^34^ 3.75 (s, 12H, OC**H**_3_), 2.36 (br m, 4H, PC**H**_2_), 1.92 (br m, 2H, PCH_2_C**H**_2_), 0.01 (s, 18H, Si(C**H**_3_)_3_); ^13^C{^1^H} (126 MHz) 161.3 (s, *p* to P), 134.9 (virtual t, ^2^*J*_CP_ = 5.9 Hz,^38^*o* to P),^35^ 121.5 (m, *J* = 73.0 Hz, *J* = 9.8 Hz, *i* to P),^39^ 113.8 (virtual t, ^3^*J*_CP_ = 5.8 Hz,^38^*m* to P),^35^ 100.3 (dd, ^2^*J*_CP*trans*_ = 147 Hz, ^2^*J*_CP*cis*_ = 21.0 Hz, ^1^*J*_CPt_ = 1133 Hz,^40^ Pt**C**≡C), 92.5 (s, ^3^*J*_CPt_ = 31.7 Hz, **C**≡CSi), 92.4 (m, *J* = 17.8 Hz,^44^^2^*J*_CPt_ = 309 Hz,^40^ PtC≡**C**), 78.2 (s, ^1^*J*_CSi_ = 87.1 Hz,^37^ C≡**C**Si), 55.2 (s, O**C**H_3_), 25.8–25.5 (m, P**C**H_2_), 19.2 (s, PCH_2_**C**H_2_), 0.1 (s, ^1^*J*_CSi_ = 56.1 Hz,^37^ Si(**C**H_3_)_3_); ^31^P{^1^H} (121 MHz) −10.8 (s, ^1^*J*_PPt_ = 2198 Hz).^40^ IR (powder film, cm^–1^) 2185/2131 (w/m, ν_C≡C_).

For **7a**, the powder slightly darkened at 202 °C and became black and liquefied at 286 °C (open capillary). Anal. Calcd for C_90_H_104_O_8_P_4_Pt_2_Si_4_ (1940.22): C, 55.71; H, 5.40. Found: C, 54.86; H, 5.59. NMR (δ/ppm, CDCl_3_): ^1^H (500 MHz) 7.52–7.48 (m, 16H, *o* to P),^34^ 6.88 (d, ^3^*J*_HH_ = 8.7 Hz, 16H, *m* to P),^34^ 3.80 (s, 24H, OC**H**_3_), 2.98 (br m, 4H, PCH_2_C**H**_2_), 2.66 (br m, 8H, PC**H**_2_), 0.07 (s, 36H, Si(C**H**_3_)_3_); ^13^C{^1^H} (126 MHz) 160.8 (s, *p* to P), 134.7 (virtual t, ^2^*J*_CP_ = 6.3 Hz,^38^*o* to P),^35^ 123.3 (virtual t, ^1^*J*_CP_ = 31.3 Hz,^38^*i* to P), 113.7 (virtual t, ^3^*J*_CP_ = 5.7 Hz,^38^*m* to P),^35^ 103.6 (t, ^2^*J*_CP_ = 15.1 Hz, Pt**C**≡C), 92.9, 92.5 (2 s, PtC≡**CC**≡CSi), 76.6 (s, C≡**C**Si), 54.9 (s, O**C**H_3_), 31.8–31.3 (m, P**C**H_2_), 26.1 (s, PCH_2_**C**H_2_), 0.0 (s, ^1^*J*_CSi_ = 56.2 Hz,^37^ Si(**C**H_3_)_3_); ^31^P{^1^H} (121 MHz) 12.0 (s, ^1^*J*_PPt_ = 2459 Hz).^40^ IR (powder film, cm^–1^) 2187/2131 (w/m, ν_C≡C_).

### (CH_2_(CH_2_P(*p*-C_6_H_4_OMe)_2_)_2_)Pt((C≡C)_2_Si(*i*Pr)_3_)_2_ (**6a**)

A round-bottom flask was charged with **4**TIPS_2_^5c^ (0.203 g, 0.167 mmol), **1a** (0.097 g, 0.18 mmol), and CH_2_Cl_2_ (20 mL) with stirring. After 2 h (a ^31^P{^1^H} NMR spectrum of an aliquot showed no remaining educt), the solvent was removed by rotary evaporation and oil pump vacuum. The brown solid was chromatographed on a silica gel column (2.5 × 30 cm, eluted first with 1:1 v/v CH_2_Cl_2_/hexanes, then 3:1 v/v CH_2_Cl_2_/hexanes). The solvents were removed from the product containing fractions by rotary evaporation and an oil pump vacuum to give **6a** as a white powder (0.180 g, 0.158 mmol, 95%) that slightly darkened at 155 °C and became black and liquefied at 226 °C (open capillary). Anal. Calcd for C_57_H_76_O_4_P_2_PtSi_2_ (1138.43): C, 60.14; H, 6.73. Found: C, 60.23; H, 6.89.

NMR (δ/ppm, CDCl_3_): ^1^H (400 MHz) 7.55–7.50 (m, 8H, *o* to P),^34^ 6.81 (d, ^3^*J*_HH_ = 8.2 Hz, 8H, *m* to P),^34^ 3.75 (s, 12H, OC**H**_3_), 2.35 (br s, 4H, PC**H**_2_), 1.92 (br m, 2H, PCH_2_C**H**_2_), 0.93 (m, 42H, C**H**(C**H**_3_)_2_); ^13^C{^1^H} (101 MHz) 161.4 (s, *p* to P), 135.1 (virtual t, ^3^*J*_CP_ = 6.0 Hz,^38^*o* to P),^35^ 121.7 (m, *J* = 72.8 Hz, *J* = 9.6 Hz, *i* to P),^39^ 113.8 (virtual t, ^3^*J*_CP_ = 5.9 Hz,^38^*m* to P),^35^ 97.9 (dd, ^2^*J*_CP*trans*_ = 147 Hz, ^2^*J*_CP*cis*_ = 21.1 Hz, Pt**C**≡C), 94.2 (s, ^3^*J*_CPt_ = 34.7 Hz,^40^**C**≡CSi), 93.5 (m, *J* = 18.0 Hz, PtC≡**C**),^44^ 74.8 (s, ^1^*J*_CSi_ = 83.2 Hz,^37^ C≡**C**Si), 55.1 (s, O**C**H_3_), 26.0–25.6 (m, P**C**H_2_), 19.4 (s, PCH_2_**C**H_2_), 18.5 (s, **C**H_3_), 11.3 (s, ^1^*J*_CSi_ = 56.6 Hz,^37^ Si**C**H(CH_3_)_2_); ^31^P{^1^H} (121 MHz) −9.1 (s, ^1^*J*_PPt_ = 2203 Hz)^40^ or in C_6_D_6_ −10.17 (s, ^1^*J*_PPt_ = 2190 Hz).^40^ IR (powder film, cm^–1^) 2180/2129 (w/m, ν_C≡C_).

### (CH_2_(CH_2_P(*p*-C_6_H_4_O*t*Bu)_2_)_2_)Pt((C≡C)_2_Si(*i*Pr)_3_)_2_ (**6b**)

A round-bottom flask was charged with **4**TIPS_2_^5c^ (0.258 g, 0.213 mmol), **1b** (0.160 g, 0.228 mmol), and CH_2_Cl_2_ (30 mL) with stirring. After 2 h (TLC showed no remaining educt), the solvent was removed by rotary evaporation and an oil pump vacuum. The solid was chromatographed on a silica gel column (3.5 × 30 cm, packed in hexanes, first eluted with hexanes, then with a CH_2_Cl_2_ gradient until pure CH_2_Cl_2_). The solvents were removed from the product containing fractions by rotary evaporation and an oil pump vacuum to give **6b** as a white solid (0.258 g, 0.197 mmol, 92%) that slightly darkened at 220 °C, liquefied at 235 °C, and became black at 239 °C (open capillary). Anal. Calcd for C_69_H_100_O_4_P_2_PtSi_2_ (1306.76): C, 63.42; H, 7.71. Found: C, 63.66; H, 7.75.

NMR (δ/ppm, CDCl_3_): ^1^H (500 MHz) 7.55–7.51 (m, 8H, *o* to P),^34^ 6.90 (d, ^3^*J*_HH_ = 8.1 Hz, 8H, *m* to P),^34^ 2.33 (br m, 4H, PC**H**_2_), 1.93–1.85 (br m, 2H, PCH_2_C**H**_2_), 1.32 (s, 36H, C(C**H**_3_)_3_), 0.94–0.84 (m, 42H, C**H**(C**H**_3_)_2_); ^13^C{^1^H} (126 MHz) 157.9 (s, *p* to P), 134.3 (virtual t, ^2^*J*_CP_ = 5.9 Hz,^38^*o* to P),^35^ 123.9 (m, *J* = 71.6 Hz, *J* = 9.8 Hz, *i* to P),^39^ 122.6 (virtual t, ^3^*J*_CP_ = 5.7 Hz,^38^*m* to P),^35^ 96.8 (dd, ^2^*J*_CP*trans*_ = 147 Hz, ^2^*J*_CP*cis*_ = 21.2 Hz, Pt**C**≡C), 94.1 (s, ^3^*J*_CPt_ = 32.8 Hz,^40^**C**≡CSi), 93.3 (m, *J* = 17.9 Hz,^44^^2^*J*_CPt_ = 307 Hz,^40^ PtC≡**C**), 79.0 (s, **C**(CH_3_)_3_), 74.7 (s, ^1^*J*_CSi_ = 82.4 Hz,^37^ C≡**C**Si), 28.8 (s, C(**C**H_3_)_3_), 26.1–25.8 (m, P**C**H_2_), 19.6 (s, PCH_2_**C**H_2_), 18.5 (s, CH(**C**H_3_)_2_), 11.3 (s, ^1^*J*_CSi_ = 56.6 Hz,^37^ Si**C**H(CH_3_)_3_); ^31^P{^1^H} (202 MHz) −9.7 (s, ^1^*J*_PPt_ = 2213 Hz).^40^ IR (powder film, cm^–1^) 2185/2131 (w/m, ν_C≡C_).

### (CH_2_(CH_2_P(*p*-C_6_H_4_SiMe_3_)_2_)_2_)Pt((C≡C)_2_Si(*i*Pr)_3_)_2_ (**6d**)

**4**TIPS_2_^5c^ (0.203 g, 0.165 mmol), **1d** (0.127 g, 0.181 mmol), and CH_2_Cl_2_ (20 mL) were combined in a procedure analogous to that for **6b**. An identical workup gave **6d** as a white powder (0.199 g, 0.152 mmol, 92%) that slightly darkened at 204 °C and became black and liquefied at 282 °C (open capillary). Anal. Calcd for C_65_H_100_P_2_PtSi_6_ (1307.06): C, 59.73; H, 7.71. Found: C, 59.96; H, 7.72.

NMR (δ/ppm, CDCl_3_): ^1^H (500 MHz) 7.69–7.65 (m, 8H, *o* to P),^34^ 7.49 (d, ^3^*J*_HH_ = 7.5 Hz, 8H, *m* to P),^34^ 2.42 (br m, 4H, PC**H**_2_), 1.99 (br m, 2H, PCH_2_C**H**_2_), 0.97–0.91 (m, 42H, C**H**(C**H**_3_)_2_), 0.26 (s, 36H, Si(C**H**_3_)_3_); ^13^C{^1^H} (126 MHz) 143.8 (s, *p* to P), 133.0 (virtual t, ^3^*J*_CP_ = 5.0 Hz,^38^*m* to P),^35^ 132.8 (virtual t, ^2^*J*_CP_ = 5.0 Hz,^38^*o* to P),^35^ 131.1 (m, *J* = 67.7 Hz, *J* = 9.7 Hz, *i* to P),^39^ 96.5 (dd, ^2^*J*_CP*trans*_ = 147 Hz, ^2^*J*_CP*cis*_ = 21.4 Hz, ^1^*J*_CPt_ = 1136 Hz,^40^ Pt**C**≡C), 94.3 (s, ^3^*J*_CPt_ = 39.9 Hz, **C**≡CSi),^40^ 93.7 (m, *J* = 17.8 Hz,^44^^2^*J*_CPt_ = 313 Hz,^40^ PtC≡**C**), 75.0 (s, ^1^*J*_CSi_ = 82.8 Hz,^37^ C≡**C**Si), 25.9–25.6 (m, P**C**H_2_), 20.2 (s, PCH_2_**C**H_2_), 18.7 (s, CH(**C**H_3_)_2_), 11.5 (s, ^1^*J*_CSi_ = 56.8 Hz,^37^ Si**C**H(CH_3_)_2_), −1.3 (s, ^1^*J*_CSi_ = 52.4 Hz,^37^ Si(**C**H_3_)_3_); ^31^P{^1^H} (202 MHz) −7.7 (s, ^1^*J*_PPt_ = 2202 Hz).^40^ IR (powder film, cm^–1^) 2184/2132 (w/m, ν_C≡C_).

### [(CH_2_(CH_2_P(*p*-C_6_H_4_OMe)_2_)_2_)Pt(C≡C)_2_]_4_·[H_2_NEt_2_^+^ Cl^–^] (**10a**·[H_2_NEt_2_^+^Cl^–^])

A Schlenk flask was charged with **3a** (0.0503 g, 0.0609 mmol), **2a** (0.0486 g, 0.0609 mmol), CuI (0.0038 g, 0.020 mmol), and THF (20 mL) with stirring and heated to 55 °C. When all solids had dissolved (ca. 10 min), HNEt_2_ (10 mL) was added, and a white precipitate rapidly formed. After 3 h, the solid was isolated by filtration, washed with hexanes (60 mL) and Et_2_O (60 mL), and dried by an oil pump vacuum (rt, 20 h) to give **10a**·[H_2_NEt_2_^+^Cl^–^] as a pale beige solid (0.0648 g, 0.0202 mmol, 66%) that darkened at 140 °C, blackened at 191 °C, and further decomposed at 255 °C (open capillary). Anal. Calcd for C_144_H_148_ClNO_16_P_8_Pt_4_ (3209.70): C, 53.84; H, 4.64; N, 0.44; Cl, 1.10. Found: C, 50.29; H, 4.37; N, 0.58; Cl, 1.09.^46^

NMR (δ/ppm, CDCl_3_): ^1^H (500 MHz) 7.52 (m, 32H, *o* to P),^34^ 6.65 (d, ^3^*J*_HH_ = 8.3 Hz, 32H, *m* to P),^34^ 3.83 (m, 4H, NC**H**_2_), 3.68 (s, 48H, OC**H**_3_), 2.27 (m, 16H, PC**H**_2_), 1.92 (br m, 8H, PCH_2_C**H**_2_), 1.47 (br m, 6H, NCH_2_C**H**_3_); ^13^C{^1^H} (126 MHz) 161.3 (s, *p* to P), 135.1 (virtual t, ^3^*J*_CP_ = 5.7 Hz,^38^*o* to P),^35^ 123.4 (m, *J* = 71.7 Hz, *J* = 8.6 Hz, *i* to P),^39^ 113.8 (virtual t, ^3^*J*_CP_ = 5.7 Hz,^38^*m* to P),^35^ 97.0 (m, Pt**C**≡C), 92.5 (dd, ^2^*J*_CP_ = 164.6 Hz, ^2^*J*_CP_ = 21.6 Hz, PtC≡**C**), 55.3 (s, O**C**H_3_), 44.6 (s, N**C**H_2_), 26.9 (m, P**C**H_2_), 20.3 (s, PCH_2_**C**H_2_), 12.6 (s, NCH_2_**C**H_3_); ^31^P{^1^H} (202 MHz) −11.3 (s, ^1^*J*_PPt_ = 2226 Hz).^40^ IR (cm^–1^, powder film): 3647 (s), 2872 (w), 2156 (w, ν_C≡C_), 1593 (m), 1499 (s), 1252 (w), 1103 (s), 823 (s), 797 (s).

### *trans-*(*p*-tol)_3_P)_2_Pt((C≡C)_2_SiEt_3_)_2_ (**4**TES_2_)

A Schlenk flask was charged with (*p*-tol_3_P)_2_PtCl_2_ (*cis*/*trans* mixture,^20^ 0.745 g, 0.852 mmol), CuI (0.080 g, 0.42 mmol), CH_2_Cl_2_ (15 mL), and HNEt_2_ (50 mL). Next, H(C≡C)_2_SiEt_3_ (0.713 g, 4.34 mmol)^47^ was added with stirring. After 3 h, the solvents were removed by rotary evaporation, and the brown residue was chromatographed on a silica gel column (2.5 × 30 cm; eluted with 3:4 v/v CH_2_Cl_2_/hexanes). The solvents were removed from the product containing fractions by rotary evaporation and an oil pump vacuum to give **4**TES_2_ as a bright yellow solid (0.755 g, 0.668 mmol, 78%) that luminesced (Figure 1), slightly darkened at 211 °C, and turned black and liquefied at 241 °C (open capillary).

NMR (δ/ppm, CDCl_3_): ^1^H (300 MHz) 7.57–7.50 (m, 12H, *o* to P),^34^ 7.16 (d, ^3^*J*_HH_ = 7.8 Hz, 12H, *m* to P),^34^ 2.35 (s, 18H, C**H**_3_*p* to P), 0.86 (t, ^3^*J*_HH_ = 7.9 Hz, 18H, CH_2_C**H**_3_), 0.42 (q, ^3^*J*_HH_ = 7.9 Hz, 12H, SiC**H**_2_); ^13^C{^1^H} (101 MHz) 140.5 (s, *p* to P), 134.9 (virtual t, ^2^*J*_CP_ = 6.4 Hz,^38^*o* to P),^35^ 128.6 (virtual t, ^3^*J*_CP_ = 5.6 Hz,^38^*m* to P),^35^ 127.5 (virtual t, ^1^*J*_CP_ = 30.6 Hz,^38^*i* to P), 105.0 (t, ^2^*J*_CP_ = 15.7 Hz, Pt**C**≡C), 95.6 (t, ^3^*J*_CP_ = 2.3 Hz, ^2^*J*_CPt_ = 266 Hz, PtC≡**C**), 93.7 (t, ^4^*J*_CP_ = 2.3 Hz, **C**≡CSi), 74.1 (t, ^5^*J*_CP_ = 1.0 Hz, C≡**C**Si), 21.3 (s, **C**H_3_*p* to P), 7.4 (s, CH_2_**C**H_3_), 4.5 (s, ^1^*J*_CSi_ = 56.2 Hz,^37^ Si**C**H_2_); ^31^P{^1^H} (121 MHz) 16.9 (s, ^1^*J*_PPt_ = 2543 Hz).^40^ IR (cm^–1^, powder film) 2187/2130 (w/m, ν_C≡C_).

### Crystallography

(A) A CH_2_Cl_2_ solution of **2a** was layered with hexanes. After 2 d, colorless prisms of **2a** were collected, and data were obtained on a Nonius Kappa CCD diffractometer as outlined in Table S4. Cell parameters were obtained from 10 frames using a 10° scan and refined with 7010 reflections. Lorentz, polarization, and absorption corrections^48^ were applied. The space group was determined from systematic absences and subsequent least-squares refinement. The structure was solved by direct methods. The parameters were refined with all data by full-matrix-least-squares on *F*^2^ using SHELXL-97.^49^ Non-hydrogen atoms were refined with anisotropic thermal parameters. The hydrogen atoms were fixed in idealized positions using a riding model. Scattering factors were taken from the literature.^50^ (B) A CH_2_Cl_2_ solution of **3a** was layered with hexanes. After 3 d, colorless prisms of **3a**·(CH_2_Cl_2_) were collected. The structure was solved and refined (cell parameters from 10 frames using a 10° scan and refined with 4566 reflections) as described for **2a** and exhibited a mirror plane perpendicular to the PPtP plane. The CH_2_Cl_2_ molecules were disordered over two positions with 50% occupancy. (C) A CH_2_Cl_2_ solution of **3d** was layered with hexanes. After 4 d, colorless needles of **3d**·(CH_2_Cl_2_) were collected. The structure was solved and refined (cell parameters from 10 frames using a 10° scan and refined with 11 230 reflections) as described for **2a**. (D) A CH_2_Cl_2_ solution of **3b** was layered with hexanes. After 1 d, colorless prisms of **3b**·(CH_2_Cl_2_)_2_ were collected, and data were obtained on a BRUKER GADDS X-ray (three circle) diffractometer as outlined in Table S3. Cell parameters were obtained from 180 frames using ω scans and refined with 12 322 reflections. Integrated intensity information for each reflection was obtained by reduction of the data frames with the program SAINT.^51^ Lorentz and polarization reduction and corrections were applied. Data were scaled, and absorption corrections were applied using the program SADABS.^52^ The structure was solved by direct methods using SHELXS-97^49^ and refined (weighted least-squares refinement on *F*^2^) using SHELXL-97 (X-Seed).^53^ Non-hydrogen atoms were refined with anisotropic thermal parameters. The hydrogen atoms were placed in idealized positions and refined using a rigid model as generated in X-Seed [C–H = 0.96 Å, *U*_iso_(H) = 1.2 × *U*_iso_(C)]. (E) A CH_2_Cl_2_ solution of **5a** was layered with hexanes. After 2 d, colorless prisms of **5a** were collected, and data were obtained on a BRUKER APEX 2 X-ray (three circle) diffractometer as outlined in Table S4. The integrated intensity information for each reflection was obtained by reduction of the data frames with the program APEX2.^54^ Cell parameters were obtained from 2400 frames using ω scans and refined with 6300 reflections. Further refinement was carried out as with **3b**. (F) A CH_2_Cl_2_ solution of **6a** was layered with Et_2_O. After 3 d, colorless needles of **6a**·(Et_2_O) were collected. The structure was solved and refined (cell parameters from 1400 frames using ω scans and refined with 14 538 reflections) as described for **5a**. (G) A CH_2_Cl_2_ solution of **4**TES_2_ was layered with hexane/MeOH. After 3 d, colorless prisms of **4**TES_2_·(CH_2_Cl_2_) were collected, and data were obtained on a Nonius Kappa CCD diffractometer as outlined in Table S4. Cell parameters were obtained from 10 frames using a 10° scan and refined with 6887 reflections. Lorentz, polarization, and absorption corrections^48^ were applied. The space group was determined from systematic absences and subsequent least-squares refinement. The structure was solved by direct methods. The parameters were refined with all data by full-matrix-least-squares on *F*^2^ using SHELXL-97.^49^ Non-hydrogen atoms were refined with anisotropic thermal parameters. The hydrogen atoms were fixed in idealized positions using a riding model. Scattering factors were taken from the literature.^50^ The molecular structure exhibits an inversion center at the platinum atom.

## Supporting Information Available

General experimental data, additional syntheses of previously reported and new compounds, additional characterization of new compounds, and tables of NMR (^31^P{^1^H}, ^13^C{^1^H}), IR, and crystallographic data (PDF)
