# Supplementary material for: Building Blocks for Molecular Polygons Based on Platinum Vertices and Polyynediyl Edges
Source: Organometallics. 2023 Feb 13;42(18):2477–91. doi: 10.1021/acs.organomet.2c00573 (PMC10852985; doi:10.1021/acs.organomet.2c00573)
Supplement: Supplementary file 1 — om2c00573_si_001.pdf [file om2c00573_si_001.pdf]

ELECTRONIC SUPPLMENTARY INFORMATION

for

# Building Blocks for Molecular Polygons Based upon Platinum Vertices and Polyynediyl Edges

**Brenna K. Collins,** [a] **Nancy Weisbach,**[a,b] **Frank Hampel,**[b] **Nattamai Bhuvanesh,**[a] **and John A. Gladysz***[a]

[a]Department of Chemistry, Texas A&M University, PO Box 30012, College Station, Texas 77842-3012, USA

[b]Institut für Organische Chemie and Interdisciplinary Center for Molecular Materials, Friedrich-Alexander-Universität Erlangen-Nürnberg, Henkestraße 42, 91054 Erlangen, Germany

E-mail: gladysz@mail.chem.tamu.edu

## Experimental Section (Part 2)

**General Data**. Reactions were conducted under dry inert atmospheres using Schlenk techniques with aerobic workups unless noted. Chemicals were treated as follows: $CH_2Cl_2$, $Et_2O$, and THF, distilled ($CaH_2$ or for ethers Na/$Ph_2CO$) or passed through a Glass Contour solvent purification system; MeOH (anhydrous, 99.8% EMD), hexanes, EtOH, $CH_2Cl_2$, and ethyl acetate, distilled by rotary evaporation or used as received (ACS grade); $HNEt_2$, distilled from $CaH_2$ (Alfa Aesar, $99^{+}$%); DMF (anhydrous, 99.8%, Alfa Aesar), $Br(CH_2)_3Br$ (98%, Alfa Aesar), $P(OEt)_3$ (98%, Alfa Aesar or Galest), $LiAlH_4$ (97%, Alfa Aesar), DIBAl-H (1.0 M in hexanes, Alfa Aesar), triphosgene (98%, Alfa Aesar), magnesium (20-100 mesh or turnings, 99.8%, Alfa Aesar), *p*-Br-$C_6H_4OMe$ (98%, Acros), *p*-$MgBrC_6H_4OMe$ (0.5 M in THF, Aldrich), $BH_3 \cdot SMe_2$ (2.0 M in THF, Acros), *p*-$BrC_6H_4O$*t*Bu) (98%, Alfa Aesar), *p*-$BrC_6H_4$*t*Bu (97%, Acros), *p*-$MgBrC_6H_4$*t*Bu (2.0 M in $Et_2O$, Aldrich), *p*-$BrC_6H_4SiMe_3$ (97%, Aldrich), $K_2PtCl_4$ (99.9%, ABCR or Pressure Chemical), CuI (99.999%, Alfa Aesar), *n*-$Bu_4N^+$ $F^-$ (1.0 M in THF, 5 wt% water, Acros), COD (97%, Acros), Celite® (Alfa Aesar), silica gel (Acros, Silicycle or Fluoroflash), and alumina ($Al_2O_3$, neutral, Brockmann I, for chromatography, 50-200 μm, Acros), used as received.

NMR spectra were recorded on modern FT spectrometers and referenced as follows (δ/ppm): $^{1}H$, residual $CHCl_3$ (7.24); $^{13}C\{^{1}H\}$, internal $CDCl_3$ (77.0); $^{31}P\{^{1}H\}$, external $H_3PO_4$ (0.00). Melting and thermal behavior was assayed using OptiMelt MPA 100 or (for TGA and DSC) Mettler-Toledo DSC-821 instruments. Microanalyses were conducted in-house (Carlo Erba EA1110 apparatus) or by Atlantic Microlab. IR spectra were recorded on a Shimadzu IRAffinity-1 spectrometer with a Pike MIRacle ATR system (diamond crystal). UV-visible and luminescence spectra were recorded on Shimadzu UV-1800 and PTI QuantaMaster spectrometers. Mass spectra were obtained using Micromass Zabspec (FAB), Applied Biosystem STR Voyager (MALDI-TOFMS), or Thermo Scientific Q Exactive Focus (APCI) instruments.

**Syntheses of Previously Reported Precursors**

**$(EtO)_2PO(CH_2)_3PO(OEt)_2$.**[s1] A round bottom flask was fitted with a distillation head, charged with $Br(CH_2)_3Br$ (43.39 g, 214.9 mmol) and $P(OEt)_3$ (100.1 g, 602.4 mmol), and heated

to 150 °C (oil bath, 20 h), during which time the EtBr generated distilled through the head. The mixture was cooled to room temperature and dried by oil pump vacuum. Distillation under reduced pressure gave $(EtO)_2PO(CH_2)_3PO(OEt)_2$ (135–140 °C, $6 \times 10^{-2}$ mbar, 53.51 g, 169.2 mmol, 79%) and the by-product $EtPO(OEt)_2$[s2] (35–40 °C, $6 \times 10^{-2}$ mbar, 28.42 g, 171.0 mmol) as air stable colorless oils.

NMR data for $(EtO)_2PO(CH_2)_3PO(OEt)_2$ (δ/ppm, $CDCl_3$): $^1H$ (300 MHz) 4.03–3.88 (m, 8H, OC**H**$_2$), 1.75–1.67 (m, 6H, PC**H**$_2$C**H**$_2$), 1.19 (t, $^3J_{HH}$ = 7.1 Hz, 12H, C**H**$_3$); $^{13}C\{^1H\}$ (75 MHz) 61.5–61.3 (m, O**C**H$_2$), 26.1 ($^1J_{CP}$ = 142 Hz, $^3J_{CP'}$ = 15.2 Hz, P**C**H$_2$), 16.4–16.2 (m, OCH$_2$**C**H$_3$), 16.1 (t, $^2J_{CP}$ = 4.7 Hz, PCH$_2$**C**H$_2$); $^{31}P\{^1H\}$ (121 MHz) 31.7 (s).

NMR data for $EtPO(OEt)_2$ (δ/ppm, $CDCl_3$): $^1H$ (300 MHz) 4.15–4.00 (m, 4H, OC**H**$_2$), 1.71 (dq, $^2J_{HP}$ = 18.1 Hz, $^3J_{HH}$ = 7.7 Hz, 2H, PC**H**$_2$CH$_3$), 1.29 (t, $^3J_{HH}$ = 7.0 Hz, 6H, POCH$_2$-C**H**$_3$), 1.13 (dt, $^3J_{HP}$ = 19.9 Hz, $^3J_{HH}$ = 7.7 Hz, 3H, PCH$_2$C**H**$_3$); $^{31}P\{^1H\}$ (121 MHz) 34.8 (s).

**$H_2P(CH_2)_3PH_2$. A** (with $LiAlH_4$).[s3] **A.** A three neck flask was fitted with a condenser and an addition funnel, and charged with $LiAlH_4$ (14.79 g, 389.7 mmol) and $Et_2O$ (350 mL). The mixture was cooled to 0 °C, and $(EtO)_2PO(CH_2)_3PO(OEt)_2$ (35.22 g, 111.4 mmol) dissolved in $Et_2O$ (40 mL) was added slowly over 2 h with stirring. After three d, the mixture was cooled to 0 °C and degassed aqueous HCl (100 mL, ~10 M) was carefully added. The organic phase was separated and the aqueous phase was washed with $Et_2O$ (2 × 50 mL). The combined organic phases were dried ($MgSO_4$) and the solvents removed by distillation. The resulting residue was distilled (145 °C) to give $H_2P(CH_2)_3PH_2$ (5.206 g, 48.18 mmol, 43%) as a colorless liquid with an obnoxious odor. **B.** (with DIBAl-H). A three neck flask was fitted with a condenser and an addition funnel, and charged with DIBAl-H (1.0 M in hexanes, 460 mL, 460 mmol). The solution was cooled to 0 °C, and $(EtO)_2PO(CH_2)_3PO(OEt)_2$ (20.01 g, 63.27 mmol) dissolved in THF (50 mL) was added dropwise with stirring over 3 h. After 16 h, the mixture was cooled to 0 °C and KOH (30 g in 200 mL degassed water) was carefully added. An aqueous solution of NaCl (43 g in 250 mL) was added and the organic phase was separated via cannula and filtered through a pad of $MgSO_4$. The solvent was removed from the filtrate and the residue distilled (145 °C) to give

$H_2P(CH_2)_3PH_2$ (3.210 g, 29.70 mmol, 47%) as a colorless liquid with an obnoxious odor.

NMR (δ/ppm, $CDCl_3$): $^1H$ (300 MHz) 2.61 (dt, $^1J_{HP}$ = 195 Hz, $^3J_{HH}$ = 7.3 Hz, 4H, $P\underline{\mathbf{H}}_2$), 1.73–1.63 (m, 2H, $PCH_2C\underline{\mathbf{H}}_2$), 1.55–1.44 (m, 4H, $PC\underline{\mathbf{H}}_2$); $^{13}C\{^1H\}$ (75 MHz) 36.0 (t, $^2J_{CP}$ = 3.0 Hz, $PCH_2\underline{\mathbf{C}}H_2$), 14.6 (dd, $J_{CP}$ = 8.7 Hz, $J_{CP'}$ = 5.4 Hz, $P\underline{\mathbf{C}}H_2$); $^{31}P\{^1H\}$ (121 MHz) −138.4 (s).

**$Cl_2P(CH_2)_3PCl_2$. A.**[s4,s5] A three neck flask was fitted with an addition funnel, cooled to −45 °C, and charged with $H_2P(CH_2)_3PH_2$ (5.739 g, 53.11 mmol) and THF (150 mL). Then triphosgene (22.06 g, 74.34 mmol) dissolved in THF (100 mL) was added dropwise over 40 min with stirring. The mixture was allowed to slowly warm to room temperature over 2 h. After 18 h, the solvent was removed and the oily residue distilled under reduced pressure (86 °C, $4 \times 10^{-2}$ mbar) to give $Cl_2P(CH_2)_3PCl_2$ (7.558 g, 30.74 mmol, 58%) as an oily liquid. **B.**[s6] A Schlenk flask was charged with $PCl_5$ (29.3088 g, 140.745 mmol) and $Et_2O$ (70 mL). Under a positive nitrogen flow, the flask was fitted with an addition funnel, which was then charged with a solution of $H_2P(CH_2)_3$-$PH_2$ (3.5628 g, 32.971 mmol) in $Et_2O$ (30 mL). The top of the funnel was fitted with a septum and venting needle to release the HCl gas evolved during the reaction. The solution was added dropwise, with stirring over the course of 15 minutes. After 24 h, the solvents were removed and the residue distilled under reduced pressure (55 °C, $10^{-1}$ mbar) to give $Cl_2P(CH_2)_3PCl_2$ (2.9138 g, 11.853 mmol, 36%) as an oily liquid.

NMR (δ/ppm, $CDCl_3$): $^1H$ (300 MHz) 2.50–2.39 (m, 4H, $PC\underline{\mathbf{H}}_2$), 2.24–2.10 (m, 2H, $PCH_2C\underline{\mathbf{H}}_2$); $^{13}C\{^1H\}$ (75 MHz) 42.7 (dd, $^1J_{CP}$ = 45.9 Hz, $^3J_{CP}$ = 8.2 Hz, $P\underline{\mathbf{C}}H_2$), 17.1 (t, $^2J_{CP}$ = 14.3 Hz, $PCH_2\underline{\mathbf{C}}H_2$); $^{31}P\{^1H\}$ (121 MHz) 190.7 (s).

## Alternative Syntheses of New Compounds

**$(p\text{-}MeOC_6H_4)_2P(CH_2)_3P(p\text{-}C_6H_4OMe)_2$ (1a;** freshly prepared as opposed to commercial Grignard reagent). A three neck flask was fitted with a condenser and an addition funnel, and charged with magnesium (1.512 g, 62.21 mmol) and THF (10 mL). The funnel was charged with $p\text{-}BrC_6H_4OMe$ (9.871 g, 52.78 mmol) dissolved in THF (20 mL), which was added dropwise over 1 h with stirring. The mixture was refluxed (oil bath, 75 °C). After 2 h, the remaining magnesium was separated and the solution was transferred to a round bottom flask fitted with an addition

funnel. The funnel was charged with $Cl_2P(CH_2)_3PCl_2$ (1.184 g, 4.816 mmol) dissolved in THF (10 mL), which was added dropwise with stirring. After 18 h, cold saturated aqueous $NH_4Cl$ (20 mL) was added and the organic phase was separated. The aqueous phase was washed with THF (2 × 30 mL) and the combined organic phases dried ($Na_2SO_4$). The solvents were removed by oil pump vacuum to give a yellow oil. Crystallization from EtOH/hexanes (–35 °C) gave **1a** as a white powder (1.404 g, 2.636 mmol, 55%). Data: see main text.

**(*p*-*t*BuC$_6$H$_4$)$_2$P(CH$_2$)$_3$P(*p*-C$_6$H$_4$*t*Bu)$_2$** (**1c**; freshly prepared as opposed to commercial Grignard reagent). A three neck flask was fitted with a condenser and an addition funnel, and charged with magnesium (1.458 g, 59.99 mmol) and THF (5 mL). The funnel was charged with *p*-BrC$_6$H$_4$*t*Bu (10.02 g, 47.02 mmol) dissolved in THF (10 mL), which was added dropwise over 1 h with stirring. The mixture was refluxed (oil bath, 75 °C). After 2 h, the remaining magnesium was separated and the solution was transferred to a round bottom flask fitted with an addition funnel. The funnel was charged with $Cl_2P(CH_2)_3PCl_2$ (1.046 g, 4.255 mmol) dissolved in THF (5 mL), which was added dropwise with stirring. After 3 d, during which time a white precipitate formed, cold saturated aqueous $NH_4Cl$ (15 mL) was added and the organic phase separated. The aqueous phase was washed with THF (3 × 50 mL) and the combined organic phases dried ($Na_2S$-$O_4$). The solvent was removed by oil pump vacuum to give a greenish oil. Crystallization from MeOH/hexanes (–20 °C) gave **1c** as a white solid (0.949 g, 1.49 mmol, 35%). Data: see main text.

### Additional Characterization of New Compounds

**(*p*-MeOC$_6$H$_4$)$_2$P(CH$_2$)$_3$P(*p*-C$_6$H$_4$OMe)$_2$ (1a).**

MS ($ESI^+$)[s7] 549 ([**M** + O + 1]$^+$, 17%), 533 ([**M** + 1]$^+$, 100%), 267 ([PAr$_2$ + Na – 1]$^+$, 46%).

**(*p*-(*t*BuOC$_6$H$_4$)$_2$P(CH$_2$)$_3$P(*p*-C$_6$H$_4$O*t*Bu))$_2$ (1b).**

MS ($ESI^+$)[s7] 717 ([**M** + O + 1]$^+$, 4%), 701 ([**M** + 1]$^+$, 100%), 351 ([PAr$_2$ + Na – 1]$^+$, 12%).

**(*p*-*t*BuC$_6$H$_4$)$_2$P(CH$_2$)$_3$P(*p*-C$_6$H$_4$*t*Bu)$_2$ (1c).**

MS ($FAB^+$, 3-NBA)[s7] 669 ([**M** + 2O + 1]$^+$, 10%), 653 ([**M** + O + 1]$^+$, 37%), 636 ([**M**]$^+$,

100%), 503 ([**M** – Ar]$^+$, 83%), 339 ([$Ar_2P(CH_2)_3$]$^+$, 20%), 297 ([$Ar_2P$]$^+$, 26%).

**(*p*-$Me_3SiC_6H_4)_2P(CH_2)_3P($*p*-$C_6H_4SiMe_3)_2$ (1d).**

MS (FAB$^+$, matrix: 3-NBA)[s7] 733 ([**M** + 2 O + 1]$^+$, 100%), 717 ([**M** + O + 1]$^+$, 43%), 701 ([**M** + 1]$^+$, 25%), 583 ([**M** + 2 O – Ar]$^+$, 11%), 567 ([**M** + O – Ar]$^+$, 20%), 551 ([**M** – Ar]$^+$, 29%).

**(*p*-(*t*$BuOC_6H_4)_2P(BH_3)(CH_2)_3P(BH_3)($*p*-$C_6H_4O$*t*Bu$))_2$ (1b·2$BH_3$).**

MS (MALDI$^+$, matrix DCTB + TFA)[s7] 728 ([**M**]$^+$, 100%), 716 ([**M** – $BH_3$ + 2]$^+$, 61%).

**($CH_2(CH_2P($*p*-$C_6H_4OMe)_2)_2)PtCl_2$ (2a).**

MS (APCI$^+$)[s7] 763 ([**M** – Cl]$^+$, 100%).

**($CH_2(CH_2P($*p*-$C_6H_4SiMe_3)_2)_2)PtCl_2$ (2d).**

DSC ($T_i/T_e/T_p/T_c/T_f$): exotherm, 72/80/113/175/175 °C. TGA: onset of mass loss, 183 °C ($T_i$).

MS (FAB$^+$, 3-NBA)[s7] 931 ([**M** – Cl]$^+$, 100%), 895 ([($Ar_2P(CH_2)_3PAr_2$)Pt]$^+$, 11%).

**($CH_2(CH_2P($*p*-$C_6H_4OMe)_2)_2)Pt((C{\equiv}C)_2H)_2$ (3a).**

MS (APCI$^+$)[s7] 826 ([**M** + 1]$^+$, 100%), 776 ([**M** – $C_4H$]$^+$, 41%), 727 ([($Ar_2P(CH_2)_3PAr_2$)-Pt]$^+$, 16%).

**($CH_2(CH_2P($*p*-*t*$BuOC_6H_4)_2)_2)Pt((C{\equiv}C)_2H)_2$ (3b).**

MS (APCI$^+$)[s7] 994 ([**M** + 1]$^+$, 73%), 944 ([**M** – $C_4H$]$^+$, 24%), 702 ([$Ar_2P(CH_2)_3PAr_2$ + 2]$^+$, 70%), 670 ([$Ar_2P(CH_2)_3PAr_2$ – 2 $CH_3$]$^+$, 100%).

**($CH_2(CH_2P($*p*-$C_6H_4$*t*$Bu)_2)_2)Pt((C{\equiv}C)_2H)_2$ (3c).**

DSC ($T_i/T_e/T_p/T_c/T_f$): exotherm, 138/180/187/193/205 °C. TGA: onset of mass loss, 204 °C ($T_i$).

UV-vis (nm, $6.24 \times 10^{-6}$ M in $CH_2Cl_2$ ($\varepsilon$, $M^{-1}cm^{-1}$)) 310 (18500), 284 (16200), 238 (72600).

MS (FAB$^+$, matrix: 3-NBA)[s7] 930 ([**M** + 1]$^+$, 29%), 880 ([**M** – $C_4H$]$^+$, 20%), 831 ([($Ar_2P$-$(CH_2)_3PAr_2$)Pt]$^+$, 100%).

**($CH_2(CH_2P($*p*-$C_6H_4SiMe_3)_2)_2)Pt((C{\equiv}C)_2H)_2$ (3d).**

DSC ($T_i/T_e/T_p/T_c/T_f$): exotherm, 183/193/198/201/219 °C. TGA: onset of mass loss, 206 °C ($T_i$).

MS ($FAB^+$, matrix: 3-NBA)[s7] 995 ([**M** + 1]$^+$, 30%), 944 ([**M** – $C_4H$ – 1]$^+$, 18%), 894 ([($Ar_2P$ $(CH_2)_3PAr_2$)Pt – 1]$^+$, 100%).

**($CH_2(CH_2P($*p*-$C_6H_4OMe)_2)_2)Pt((C≡C)_2SiMe_3)_2$ (5a).**

MS ($APCI^+$)[s7] 970 ([**M**]$^+$, 100%), 849 ([**M** – $C_4$TMS + 1]$^+$, 25%), 727 ([($Ar_2P(CH_2)_3$-$PAr_2$)Pt]$^+$, 35%).

***trans,trans*-$(Me_3Si(C≡C)_2)_2Pt(($*p*-$MeOC_6H_4)_2P(CH_2)_3P($*p*-$C_6H_4OMe)_2)_2Pt((C≡C)_2SiMe_3)_2$.**

UV-vis (nm, 1.34 × $10^{-5}$ M in $CH_2Cl_2$ (ε, $M^{-1}cm^{-1}$)) 318 (14100) 337 (39600).

MS ($APCI^+$)[s7] 1941 ([**M** + 1]$^+$, 29%), 1455 ([**M** – 4 $C_4$TMS – 1]$^+$, 5%).

**($CH_2(CH_2P($*p*-$C_6H_4OMe)_2)_2)Pt((C≡C)_2Si($*i*Pr$)_3)_2$ (6a).**

DSC ($T_i/T_e/T_p/T_c/T_f$): exotherm, 108/129/133/135/149 °C; endotherm, 173/186/192/201 /211 °C; exotherm, 213/237/238/238/240 °C. TGA: onset of mass loss, 217 °C ($T_i$).

MS ($FAB^+$, matrix: 3-NBA)[s7] 1138 ([**M**]$^+$, 11%), 932 ([**M** – $C_4$TIPS]$^+$, 11%), 727 ([($Ar_2P(CH_2)_3PAr_2$)Pt]$^+$, 100%).

**($CH_2(CH_2P($*p*-$C_6H_4$*t*BuO$)_2)_2)Pt((C≡C)_2Si($*i*Pr$)_3)_2$ (6b).**

MS ($APCI^+$)[s7] 1307 ([**M**]$^+$, 100%).

**($CH_2(CH_2P($*p*-$C_6H_4SiMe_3)_2)_2)Pt((C≡C)_2Si($*i*Pr$)_3)_2$ (6d).**

MS ($APCI^+$)[s7] 1308 ([**M** + 1]$^+$, 100%).

**[($CH_2(CH_2P($*p*-$C_6H_4OMe)_2)_2)Pt(C≡C)_2]_4·[H_2NEt_2^+ Cl^-]$ (10a·$[H_2NEt_2^+ Cl^-]$).**

MS: $MALDI^+$ (matrix DCTB)[s7] 3102 ([**10a**]$^+$, 100%).

***trans*-((*p*-tol)$_3P)_2Pt((C≡C)_2SiEt_3)_2$ (4TES$_2$).**

DSC ($T_i/T_e/T_p/T_c/T_f$): exotherm, 104/117/132/143/157 °C. TGA: onset of mass loss, 242 °C ($T_i$). Calcd for $C_{62}H_{72}P_2PtSi_2$ (1129.43): C, 65.87; H, 6.42; Found: C, 65.67; H, 6.44.

UV-vis (1.37 × $10^{-5}$ M in $CH_2Cl_2$ (ε, $M^{-1}cm^{-1}$)) 319 (9500), 339 (26500).

MS ($FAB^+$, matrix: 3-NBA)[s7] 1130 ([M]$^+$, 31%), 966 ([M – $C_4$TES]$^+$, 25%), 803 ([((*p*-tol)$_3P)_2$Pt]$^+$, 100%).

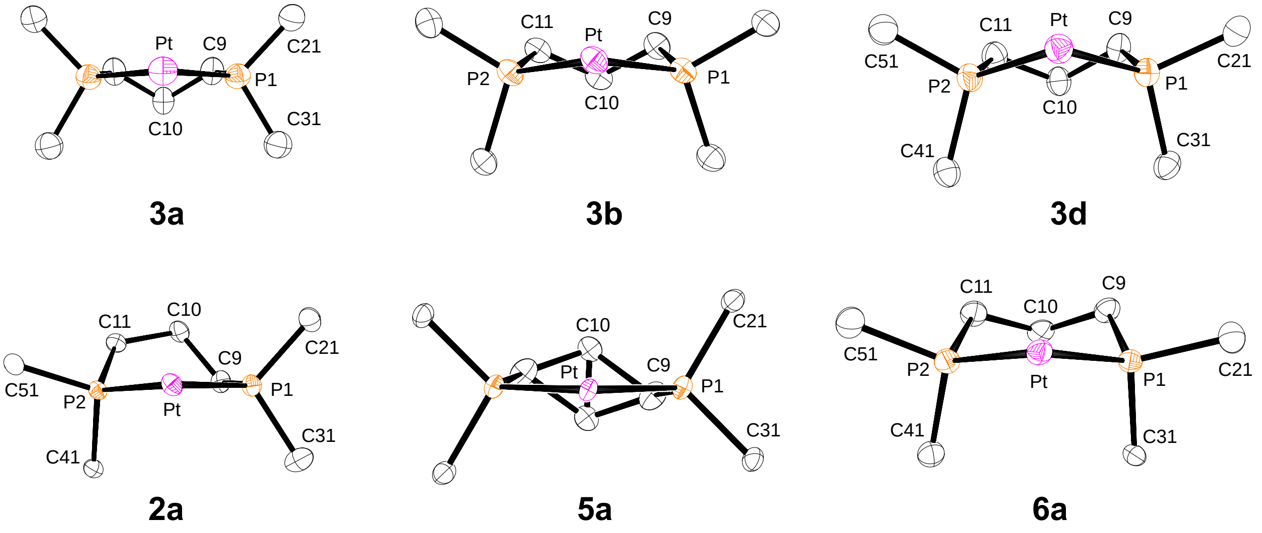


**Figure s1.** Thermal ellipsoid plots (50% probability level) highlighting the six-membered chelate rings of the cryst-allographically characterized complexes (both disordered $CH_2$ groups depicted in **5a**). Selected torsion angles (°; **2a**/**3a**/**3b**/**3d**/**5a**/**6a**): C9-P1-P2-C11, –32.5/0.0/2.2/3.2/–14.4/2.0; Pt-P1-C9-C10, 51.4/–41.7/–53.5/–59.3/–14.2/ –60.4; Pt-P2-C11-C10, 30.8/48.4/48.3/51.8/45.7/60.0.

## References


(s1) Kosolapoff, G. M. Isomerization of Tri-alkyl Phosphites. II. Reaction between Triethyl Phosphite and Trimethylene Bromide. *J. Am. Chem. Soc.* **1944**, *66*, 1511-1512.

(s2) Vugts, D. J.; Koningstein, M. M.; Schmitz, R. F.; de Kanter, F. J. J.; Groen, M. B.; Orru, R. V. A. Multicomponent Synthesis of Dihydropyrimidines and Thiazines. *Chem. Eur. J.* **2006**, *12*, 7178-7189.

(s3) Maier, L. Organische Phosphorverbindungen XXII. Darstellung und Eigenschaften von diprimären α,ω-Bis-phosphino-alkanen. *Helv. Chim. Acta* **1966**, *49*, 842-851.

(s4) Humphreys, A. S.; Filipovska, A.; Berners-Price, S. J.; Koutsantonis, G. A.; Skelton, B. W.; White, A. H. Gold(I) chloride adducts of 1,3-bis(di-2-pyridylphosphino)propane: synthesis, structural studies and antitumour activity. *Dalton Trans.* **2007**, 4943-4950.

(s5) Berven, B. M.; Koutsantonis, G. A. Highly Fluorous Bidentate Phosphines. *Synthesis* **2008**, *16*, 2626-2630.

(s6) Tavtorkin, A. N.; Toloraya, S. A.; Nifant'ev, E. E.; Nifant'ev, I. E. A new method for the synthesis of dichlorophosphines. *Tetrahedron Lett.* **2011**, *52*, 824-825.

(s7) The most intense peak of the isotope envelope is given.

**Table s1.** $^{31}P\{^1H\}$ NMR (δ/ppm, $CDCl_3$; *J* values in Hz) and key IR ($cm^{-1}$, powder film) data.

| compound | $^{31}P\{^1H\}$ NMR [$^1J_{PPt}$] [a] | IR ($\nu_{\equiv CH}$ or $\nu_{C\equiv C}$) |
|---|---|---|
| **1a** | −19.9 | – |
| **1b** | −19.5 | – |
| **1c** | −19.6 | – |
| **1d** | −16.6 | – |
| **1b·2BH$_3$** | 13.6 | – |
| **2a** | −7.0 [3428] | – |
| **2d** | −4.1 [3416] | – |
| **3a** | −9.7 [2211] | **3275**/2149 (w/m) |
| **3b** | −9.6 [2202] | **3291**/2151 (w/m) |
| **3c** | −9.0 [2196] | **3312**/2151 (m/s) |
| **3d** | −6.6 [2191] | **3315**/**3289**/2151 (w/w/m) |
| **4TES$_2$** | 16.9 [2543] | 2187/2130 (w/m) |
| **5a** | −10.8 [2198] | 2185/2131 (w/m) |
| **6a** | −9.1 [2203] | 2180/2129 (w/m) |
| **6b** | −9.7 [2213] | 2185/2131 (w/m) |
| **6d** | −7.7 [2202] | 2184/2132 (w/m) |
| **7a** | 12.0 [2459] | 2187/2131 (w/m) |
| **10a** | −11.3 [2226] | 2156 (w) |

[a] Disregarding the satellites, all signals are singlets.

**Table s2.** $^{13}C\{^1H\}$ NMR data (δ/ppm, $CDCl_3$) for the diphosphines **1** and platinum adducts.[a]

| compound | *i* to P | *o* to P | *m* to P | *p* to P | $PCH_2$ | $PCH_2\underline{CH_2}$ | *para* groups |
|---|---|---|---|---|---|---|---|
| **1a** | 129.6 | 134.0 | 114.0 | 159.9 | 30.2 | 22.3 | 55.1 |
| **2a** | 119.7 | 135.0 | 114.0 | 161.7 | 25.6 | 18.6 | 55.3 |
| **3a** | 121.5 | 135.0 | 114.0 | 161.5 | 25.9 | 19.6 | 55.3 |
| **5a** | 121.5 | 134.9 | 113.8 | 161.3 | 25.7 | 19.2 | 55.2 |
| **6a** | 121.7 | 135.1 | 113.8 | 161.4 | 25.8 | 19.4 | 55.1 |
| **7a** | 123.3 | 134.7 | 113.7 | 160.8 | 31.6 | 26.1 | 54.9 |
| **10a** | 123.4 | 135.1 | 113.8 | 161.3 | 26.9 | 20.3 | 55.3 |
| **1b** | 132.3 | 133.4 | 123.6 | 156.0 | 30.3 | 22.5 | 78.6, 28.9[b] |
| **3b** | 123.5 | 134.1 | 122.9 | 158.0 | 25.7 | 19.4 | 79.4, 28.9[b] |
| **6b** | 123.9 | 134.3 | 122.6 | 157.9 | 25.9 | 19.6 | 79.0, 28.8[b] |
| **1c** | 135.1 | 132.5 | 125.3 | 151.4 | 29.9 | 22.8 | 34.6, 31.3[b] |
| **3c** | 126.9 | 133.3 | 125.3 | 154.0 | 25.2 | 19.6 | 34.7, 31.0[b] |
| **1d** | 139.0 | 131.9 | 133.2 | 140.8 | 29.5 | 22.7 | −1.2 |
| **2d** | 129.0 | 132.7 | 133.2 | 144.7 | –[c] | –[c] | −1.3 |
| **3d** | 130.6 | 132.6 | 133.1 | 144.1 | 24.9 | 19.7 | −1.5 |
| **6d** | 131.1 | 132.8 | 133.0 | 143.8 | 25.8 | 20.2 | −1.3 |

[a] Signals were assigned as described in footnotes to the experimental section. [b] This signal is for the $CH_3$ group. [c] This signal was not observed.

**Table s3.** General crystallographic data for $CH_2Cl_2$ monosolvates of **3a**,**b**,**d**.

| | **3a**·($CH_2Cl_2$) | **3b**·($CH_2Cl_2$)$_2$ | **3d**·($CH_2Cl_2$) |
|---|---|---|---|
| empirical formula | $C_{40}H_{37}Cl_2O_4P_2Pt$ | $C_{53}H_{64}Cl_4O_4P_2Pt$ | $C_{48}H_{62}Cl_2P_2PtSi_4$ |
| formula weight | 909.63 | 1163.87 | 1079.27 |
| temperature [K] | 173(2) | 173(2) | 173(2) |
| wavelength [Å] | 0.71073 | 0.71069 | 0.71073 |
| crystal system | orthorhombic | monoclinic | monoclinic |
| space group | *Pnma* | *C*2/*c* | *Cc* |
| unit cell dimensions | | | |
| *a* [Å] | 15.5619(4) | 37.155(6) | 15.0633(4) |
| *b* [Å] | 22.9780(7) | 12.3950(5) | 36.3822(8) |
| *c* [Å] | 10.9144(2) | 28.180 | 11.5153(3) |
| α [°] | 90 | 90 | 90 |
| β [°] | 90 | 123.80(5) | 123.509(1) |
| γ [°] | 90 | 90 | 90 |
| volume [$Å^3$] | 3902.8(2) | 10785(3) | 5261.9(2) |
| *Z* | 4 | 8 | 4 |
| $\rho_{calc}$ [$Mg/m^3$] | 1.548 | 1.434 | 1.362 |
| μ [$nm^{-1}$] | 3.853 | 2.901 | 2.950 |
| F(000) | 1804 | 4720 | 2192 |
| crystal size [mm] | 0.25 × 0.20 × 0.15 | 0.30 × 0.10 × 0.10 | 0.20 × 0.20 × 0.02 |
| $\Theta$ range [°] | 2.07 to 27.48 | 2.25 to 26.26 | 2.33 to 27.50 |
| index ranges | $-20 \le h \le 19$<br>$-29 \le k \le 29$<br>$-14 \le l \le 14$ | $-48 \le h \le 48$<br>$-16 \le k \le 14$<br>$-36 \le l \le 36$ | $-19 \le h \le 19$<br>$-46 \le k \le 47$<br>$-14 \le l \le 14$ |
| reflections collected | 8369 | 61804 | 11243 |
| independent reflections | 4566 [R(int) = 0.0414] | 12322 [R(int) = 0.0363] | 11230 [R(int) = 0.0249] |
| data/restraints/parameters | 4566/6/236 | 12322/41/602 | 11230/2/515 |
| goodness-of-fit on $F^2$ | 1.032 | 1.077 | 1.025 |
| final R indices [I>2σ(I)] | $R1 = 0.0522$, $wR^2 = 0.1414$ | $R1 = 0.0279$, $wR^2 = 0.0713$ | $R1 = 0.0378$, $wR^2 = 0.0933$ |
| *R* indices (all data) | $R1 = 0.0821$, $wR^2 = 0.1583$ | $R1 = 0.0376$, $wR^2 = 0.0794$ | $R1 = 0.0446$, $wR^2 = 0.0971$ |
| largest diff. peak/hole [$eÅ^{-3}$] | 2.753/–1.952 | 1.89/–0.63 | 1.211/–1.679 |

**Table s4.** General crystallographic data for **2a**, **5a**, **6a**, and **4**TES$_2$ or solvates thereof.

| | **2a** | **5a** | **6a**·(Et$_2$O) | **4**TES$_2$·(CH$_2$Cl$_2$) |
|---|---|---|---|---|
| empirical formula | $C_{31}H_{34}Cl_2O_4P_2Pt$ | $C_{45}H_{52}O_4P_2PtSi_2$ | $C_{61}H_{86}O_5P_2PtSi_2$ | $C_{64}H_{76}Cl_4P_2PtSi_2$ |
| formula weight | 798.51 | 970.07 | 1212.50 | 1300.25 |
| temperature [K] | 173(2) | 110(2) | 110(2) | 173(2) |
| wavelength [Å] | 0.71073 | 0.71073 | 0.71073 | 0.71073 |
| crystal system | triclinic | monoclinic | monoclinic | triclinic |
| space group | $P\bar{1}$ | $C2/c$ | $P2_1$ | $P\bar{1}$ |
| unit cell dimensions | | | | |
| $a$ [Å] | 8.2245(1) | 22.5731(9) | 15.1670(8) | 11.3680(3) |
| $b$ [Å] | 10.6118(2) | 9.0549(4) | 12.6960(7) | 13.1210(2) |
| $c$ [Å] | 18.1740(3) | 22.6198(9) | 16.7980(13) | 13.1938(2) |
| α [°] | 84.488(1) | 90 | 90 | 118.5420(1) |
| β [°] | 86.488(1) | 109.066(1) | 108.398(2) | 90.2860(1) |
| γ [°] | 75.855(1) | 90 | 90 | 110.5320(1) |
| volume [$Å^3$] | 1529.79(4) | 4369.8(3) | 3096.3(3) | 1582.96(5) |
| $Z$ | 2 | 4 | 2 | 1 |
| $\rho_{calc}$ [$Mg/m^3$] | 1.734 | 1.475 | 1.312 | 1.364 |
| μ [$nm^{-1}$] | 4.901 | 3.380 | 2.421 | 2.510 |
| F(000) | 788 | 1960 | 1256 | 664 |
| crystal size [mm] | 0.25 × 0.20 × 0.20 | 0.10 × 0.08 × 0.07 | 0.20 × 0.10 × 0.02 | 0.30 × 0.20 × 0.10 |
| $\Theta$ range [°] | 1.13 to 27.58 | 1.91 to 30.00 | 2.55 to 26.26 | 1.93 to 27.49 |
| index ranges | $-10 \leq h \leq 10$ | $-31 \leq h \leq 30$ | $-20 \leq h \leq 20$ | $-14 \leq h \leq 14$ |
| | $-13 \leq k \leq 13$ | $-12 \leq k \leq 12$ | $-16 \leq k \leq 16$ | $-17 \leq k \leq 16$ |
| | $-23 \leq l \leq 23$ | $-31 \leq l \leq 31$ | $-22 \leq l \leq 22$ | $-17 \leq l \leq 17$ |
| reflections collected | 13182 | 38722 | 55284 | 13675 |
| independent reflections | 7010 [R(int) = 0.0206] | 6300 [R(int) = 0.0785] | 14538 [R(int) = 0.0449] | 7241 [R(int) = 0.0199] |
| data/restraints/parameters | 7010/0/361 | 6300/0/254 | 14538/17/658 | 7241/6/338 |
| goodness-of-fit on $F^2$ | 1.058 | 1.044 | 1.030 | 1.035 |
| final R indices [I>2σ(I)] | $R1 = 0.0248$, $wR^2 = 0.0590$ | $R1 = 0.0256$, $wR^2 = 0.0632$ | $R1 = 0.0300$, $wR^2 = 0.0583$ | $R1 = 0.0260$, $wR^2 = 0.0609$ |
| $R$ indices (all data) | $R1 = 0.0277$, $wR^2 = 0.0621$ | $R1 = 0.0265$, $wR^2 = 0.0639$ | $R1 = 0.0405$, $wR^2 = 0.0610$ | $R1 = 0.0263$, $wR^2 = 0.0611$ |
| largest diff. peak/hole [$eÅ^{-3}$] | 1.321/–1.175 | 1.458/–1.955 | 1.81/–0.49 | 1.033/–1.220 |
